# Supplementary material for: Microtubules Deform the Nuclear Membrane and Disrupt Nucleocytoplasmic Transport in Tau-Mediated Frontotemporal Dementia
Source: Cell Rep. 2019 Jan 15;26(3):582–593.e5. doi: 10.1016/j.celrep.2018.12.085 (PMC6335264; doi:10.1016/j.celrep.2018.12.085)
Supplement: Document S1. Figures S1–S5 [file mmc1.pdf]

Cell Reports, Volume *26*

Supplemental Information

Microtubules Deform the Nuclear Membrane and Disrupt Nucleocytoplasmic Transport in Tau-Mediated Frontotemporal Dementia

Francesco Paonessa, Lewis D. Evans, Ravi Solanki, Delphine Larrieu, Selina Wray, John Hardy, Stephen P. Jackson, and Frederick J. Livesey

Figure S1

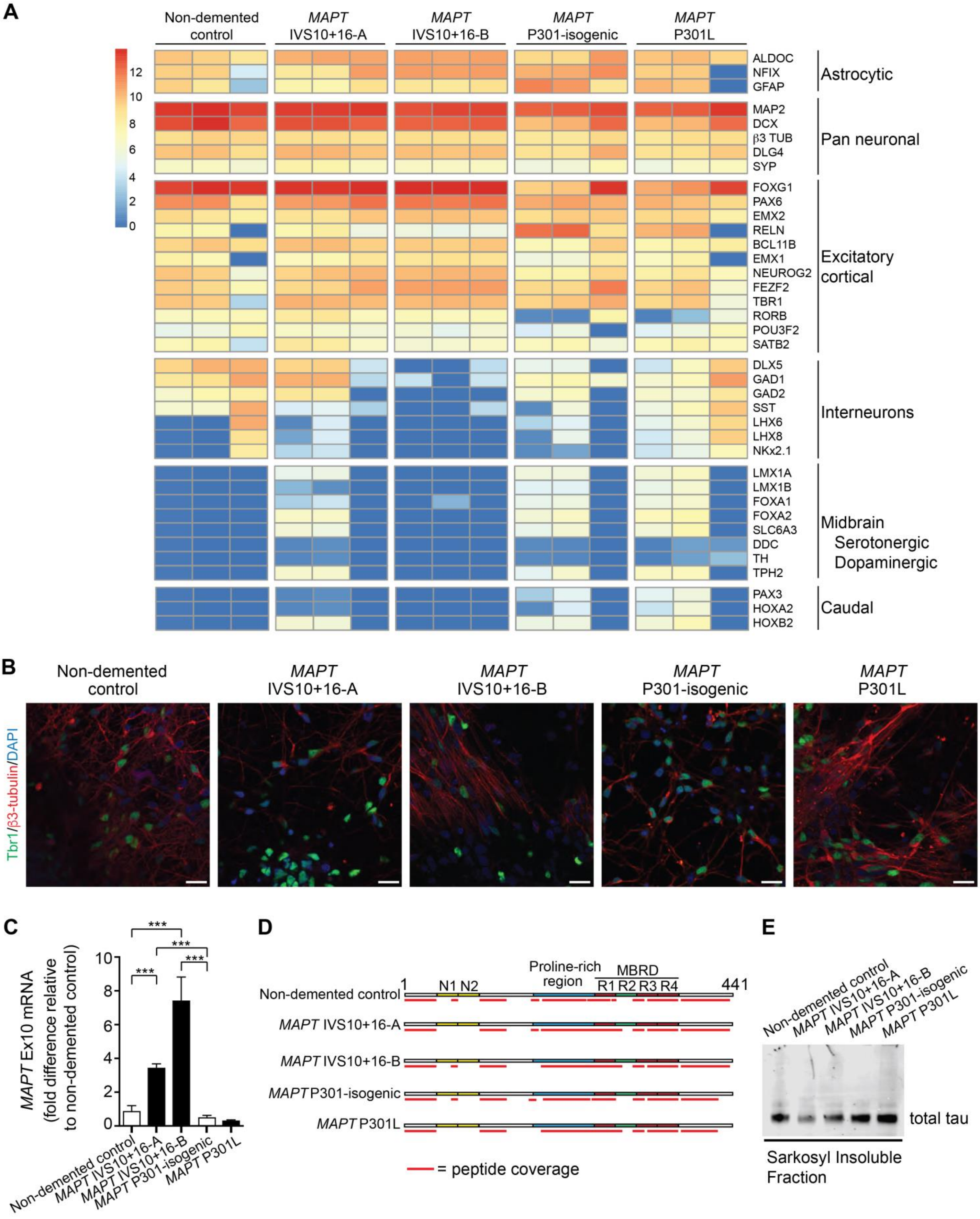
A
Non-demented control
MAPT IVS10+16-A
MAPT IVS10+16-B
MAPT P301-isogenic
MAPT P301L
12
10
8
6
4
2
0
ALDOC
NFIX
GFAP
Astrocytic
MAP2
DCX
β3 TUB
DLG4
SYP
Pan neuronal
FOXG1
PAX6
EMX2
RELN
BCL11B
EMX1
NEUROG2
FEZF2
TBR1
RORB
POU3F2
SATB2
Excitatory cortical
DLX5
GAD1
GAD2
SST
LHX6
LHX8
NKx2.1
Interneurons
LMX1A
LMX1B
FOXA1
FOXA2
SLC6A3
DDC
TH
TPH2
Midbrain
Serotonergic
Dopaminergic
PAX3
HOXA2
HOXB2
Caudal
B
Non-demented control
MAPT IVS10+16-A
MAPT IVS10+16-B
MAPT P301-isogenic
MAPT P301L
Tbr1/β3-tubulin/DAPI
C
MAPT Ex10 mRNA (fold difference relative to non-demented control)
10
8
6
4
2
0
***
***
***
***
Non-demented control
MAPT IVS10+16-A
MAPT IVS10+16-B
MAPT P301-isogenic
MAPT P301L
D
1
N1 N2
Proline-rich region
MBRD
R1 R2 R3 R4
441
Non-demented control
MAPT IVS10+16-A
MAPT IVS10+16-B
MAPT P301-isogenic
MAPT P301L
= peptide coverage
E
Non-demented control
MAPT IVS10+16-A
MAPT IVS10+16-B
MAPT P301-isogenic
MAPT P301L
total tau
Sarkosyl Insoluble Fraction

Related to Figure 1; Figure S1. Generation and characterization of human iPSC-derived neurons from familial FTD-*MAPT*.

(A) Heat map showing the relative expression of 38 genes (clustered by cell type) from FTD-MAPT neurons (*MAPT* IVS10+16-A/B and *MAPT* P301L) and non-demented and MAPT P301 isogenic control neurons (iPSC-derived neurons at 120 DIV; three biological replicates). **(B)** Immunofluorescences of iPSC-derived cerebral cortex excitatory neurons generated from control (non-demented control and *MAPT* P301-isogenic) and familial FTD-*MAPT* (*MAPT* IVS10+16-A/B and *MAPT* P301L), expressing the cortical layer 6 transcription factor Tbr1 (green) and β3-tubulin (red). DNA counterstain (DAPI, blue) is also shown. Scale bar = 20 μm. **(C)** qRT-PCR showing the relative abundance of *MAPT* mRNA containing the exon 10 (encoding for the additional microtubule binding repeat). FDT-*MAPT* (red) neurons are shown with respect to their respective controls (green). GAPDH and RPS9 were used as housekeeping genes (one-way ANOVA followed by Tukey's test, *** = P<0.001; error bar represents s.e.m.; n = 3 independent experiments). **(D)** Schematic of mass spectrometry peptide coverage (red line) for tau protein following immunoprecipitation of neuronal cell lysates from FTD-*MAPT* and control neurons (same genotypes as A; 120 DIV). 1 and 441 represent the NH- and COOH- protein extremities respectively. The position of alternatively spliced N1 and N2 domains (yellow), the proline rich domain (blue). The microtubule binding region domain (MBRD) has four microtubule binding regions R1,3 and 4 are shown in green, while the alternatively spliced R2 is shown in red. **(E)** Total tau detected in the sarkosyl-insoluble fraction across the indicated genotypes. No significant difference was detected in the amount of tau detected from neurons of each genotype.

Figure S2

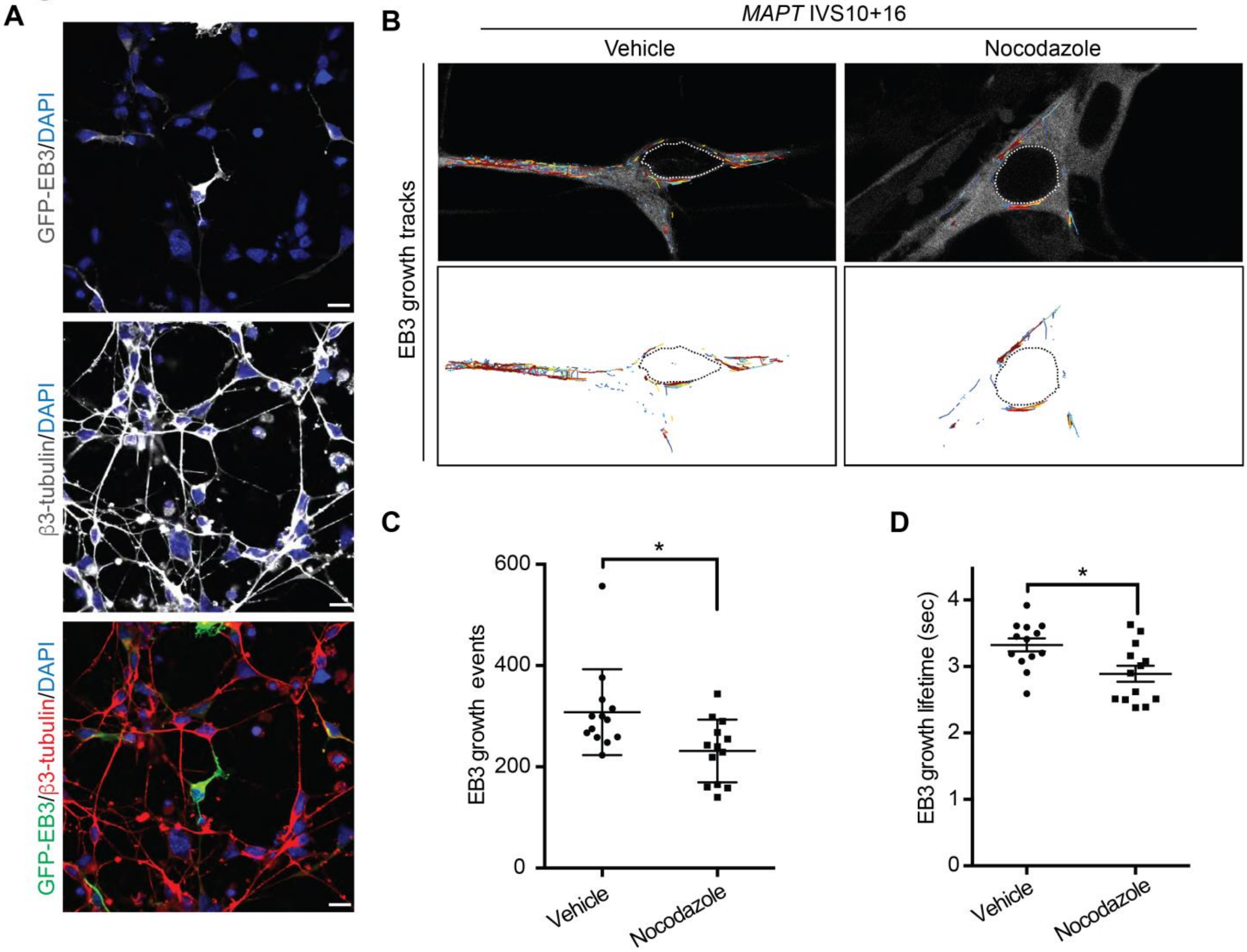
A
GFP-EB3/DAPI
β3-tubulin/DAPI
GFP-EB3/β3-tubulin/DAPI
B
MAPT IVS10+16
Vehicle
Nocodazole
EB3 growth tracks
C
EB3 growth events
600
400
200
0
*
Vehicle
Nocodazole
D
EB3 growth lifetime (sec)
4
3
2
1
0
*
Vehicle
Nocodazole

Related to Figure 2; Figure S2. Expression of GFP-EB3 recombinant protein in human iPSC-derived neurons.

(A) Immunofluorescence of iPSC-derived neurons (non-demented control; 120DIV) expressing GFP-EB3 protein, following (48 hr) lentivirus transfection. Left panel shows β3-tubulin (grey) and DAPI (blue). Middle panel shows β3-tubulin (grey) and DAPI (blue). Right panel shows merge of GFP-EB3 (green), β3-tubulin (red) and DAPI (blue). Scale bar = 10 µm. **(B)** Total microtubule trajectories (200-second interval; identified using plusTip track software) demonstrate reduction of EB3 comets after nocodazole treatment in FTD-MAPT neurons (MAPT IVS10+16-A; 120 DIV), overlaid on stills from GFP-EB3 live imaging (grey), white dotted line indicates the nucleus. **(C)** Quantification of reduction in EB3 growth events and **(D)** EB3 growth life time after two hours of nocodazole treatment. Pair-wise comparison of the vehicle and nocodazole treated neurons was performed using Student's *t* test; *=$P<0.05$; error bar represents s.e.m.; n >10 cells).

Figure S3

A

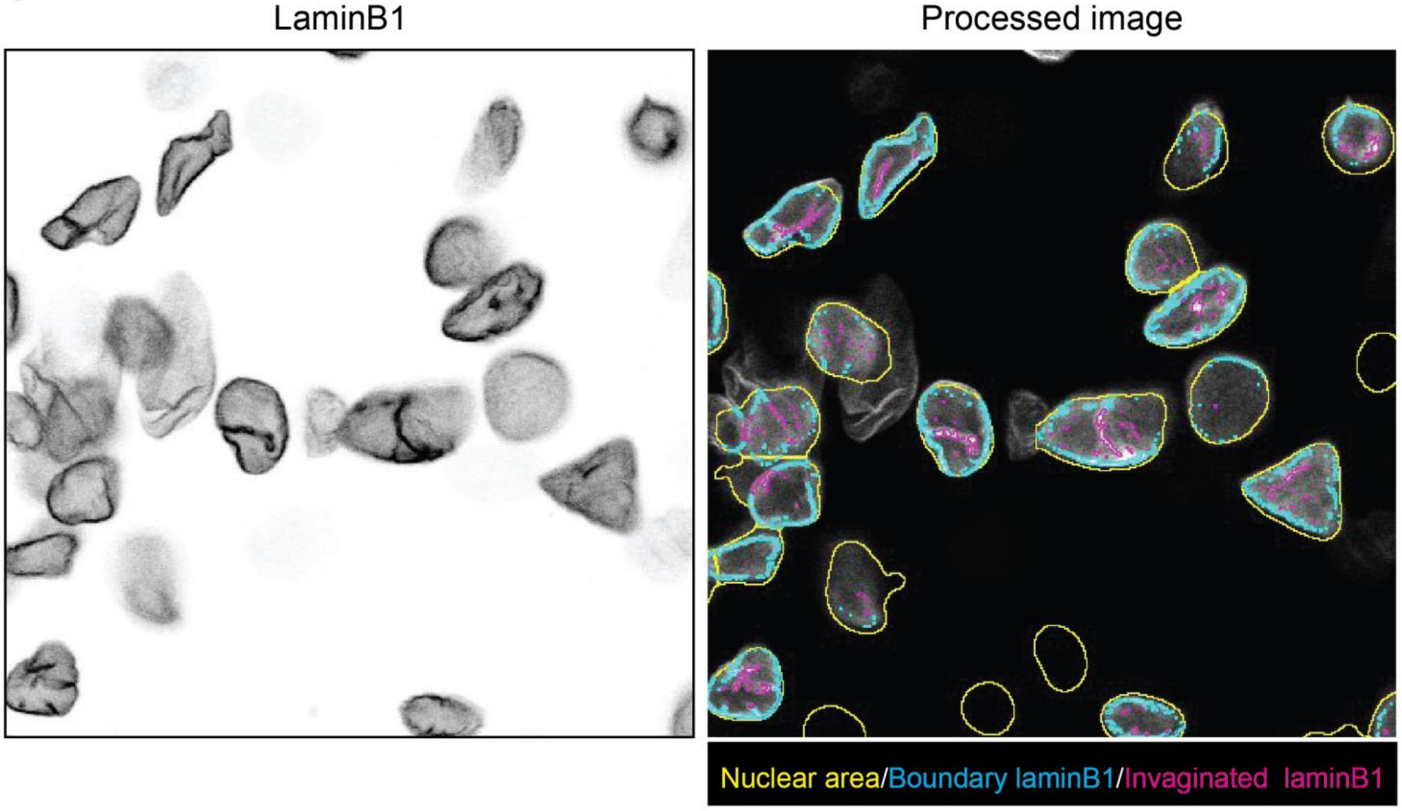

Boundary laminB1 + Invaginated laminB1 = Total laminB1

Invaginated laminB1 proportion = Invaginated laminB1/Total laminB1

B

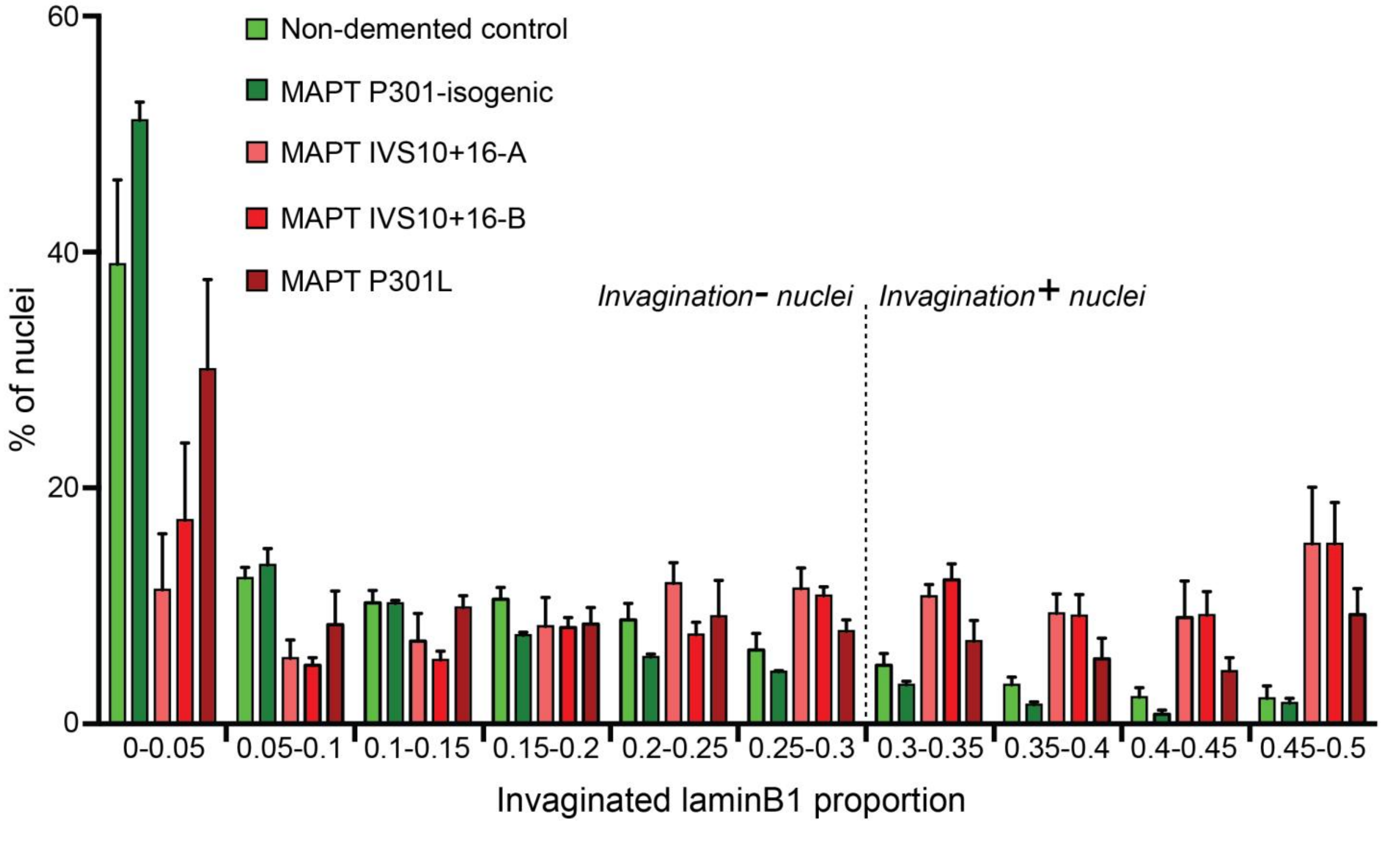

Related to Figure 3; Figure S3. Analysis used to quantify nuclear invaginations in iPSC-derived neurons.

(A) Immunofluorescence of iPSC-derived neurons (*MAPT* P301L; 120DIV), left panel shows LaminB1 (black) and right panel shows processing algorithm performed using the Fiji software. Distinct nuclei are identified by yellow boundaries. LaminB1 signal was assigned as either proximal to the nuclear boundary (cyan) or invaginated (i.e., within the nucleus; magenta). The invaginated laminB1 proportion is calculated from the invaginated laminB1 divided by the total laminB1 signal per nucleus. **(B)** Percentage of nuclei with binned invaginated laminB1 proportions (0.05 intervals) was used to threshold nuclei that are positive for invagination. A distinct population is evident >0.25 invaginated laminB1, this was assigned as invagination positive.

Figure S4

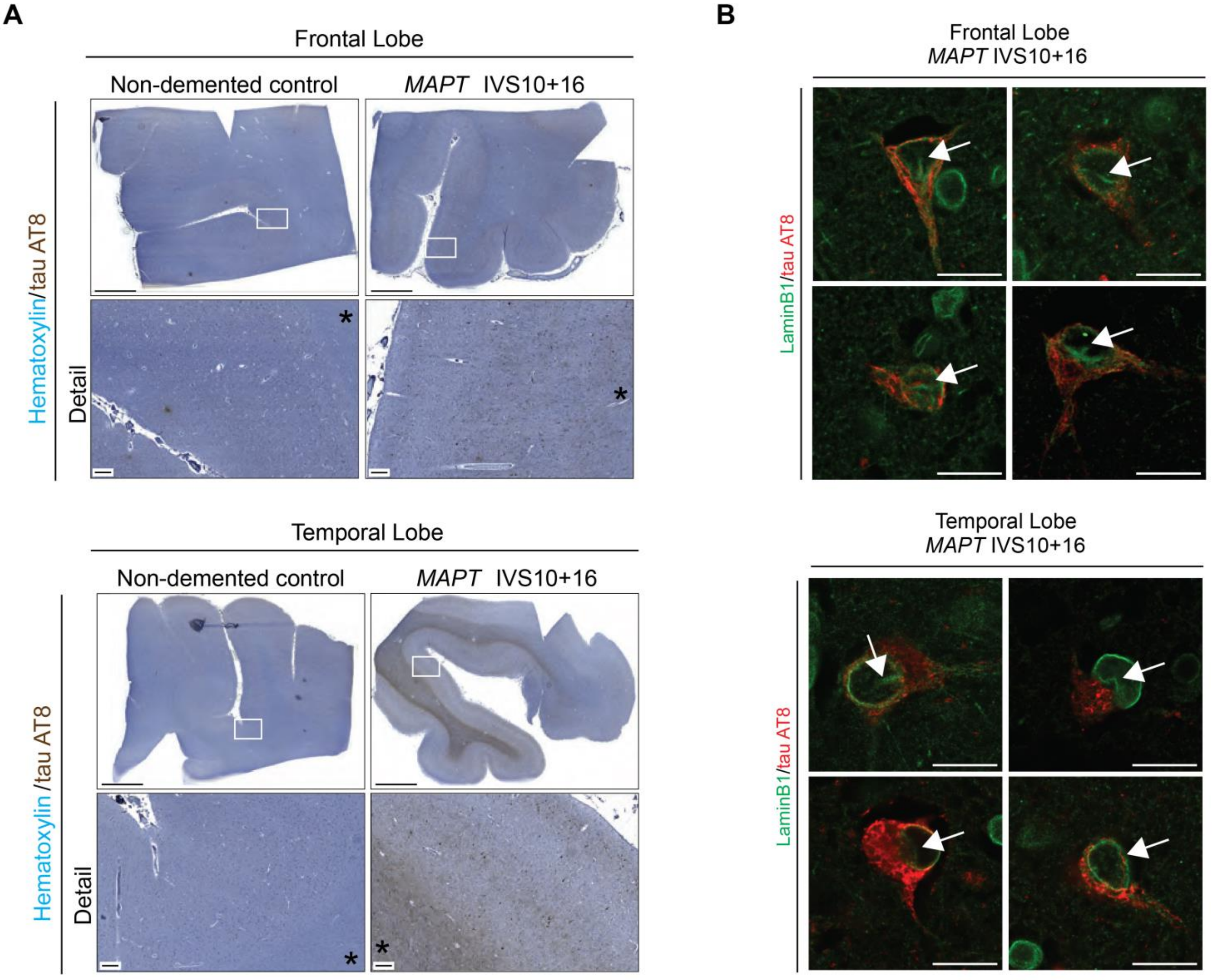
A
Frontal Lobe
Non-demented control
MAPT IVS10+16
Hematoxylin/tau AT8
Detail
Temporal Lobe
Non-demented control
MAPT IVS10+16
Hematoxylin /tau AT8
Detail
B
Frontal Lobe
MAPT IVS10+16
LaminB1/tau AT8
Temporal Lobe
MAPT IVS10+16
LaminB1/tau AT8

Related to Figure 5; Figure S4. Phosphorylated disease associated tau is present in *post-mortem* sections from frontal and temporal lobe of FTD-*MAPT*.

(A) 3,3′-Diaminobenzidine (DAB) staining for phosphorylated tau AT8 (brown) in frontal and temporal lobe of subjects carrying MAPT IVS10+16 mutation or non-demented controls (age matched), hematoxylin (blue) was used as nuclear counterstain. Lower panels, detail from white boxes in upper panels. Asterisk indicates white matter position. Scale bars; upper panel = 400 µm, lower panel = 4 µm. **(B)** Confocal images of representative post-mortem neurons from frontal and temporal lobe of subjects carrying MAPT IVS10+16 mutation showing nuclear lamina (green) invaginations (white arrows) in phosphorylated tau (AT8; pS202/pT205; red) containing neurons. Scale bar = 20 µm.

Figure S5

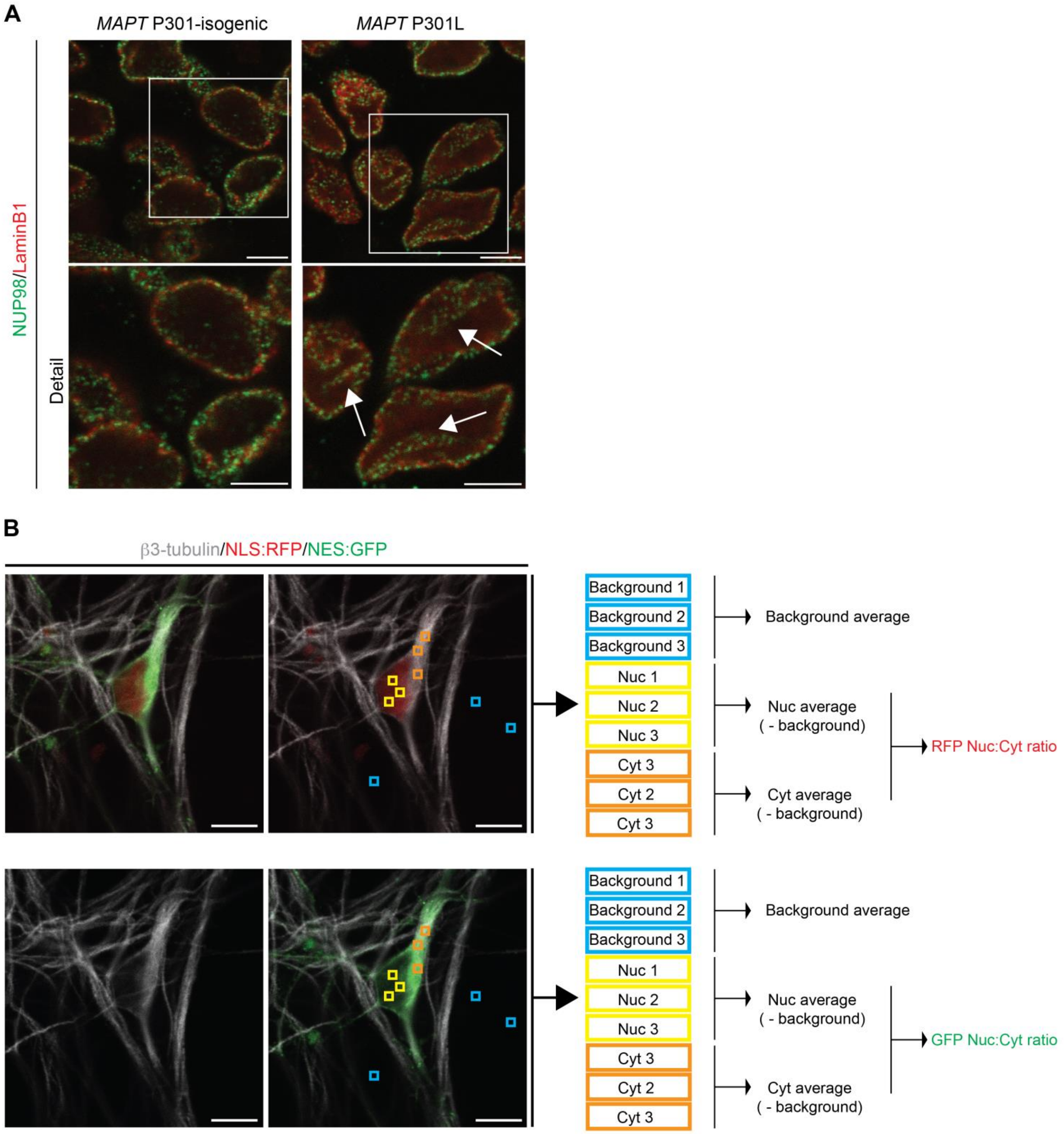
A
MAPT P301-isogenic
MAPT P301L
NUP98/LaminB1
Detail
B
β3-tubulin/NLS:RFP/NES:GFP
Background 1
Background 2
Background 3
Background average
Nuc 1
Nuc 2
Nuc 3
Nuc average (- background)
Cyt 3
Cyt 2
Cyt 3
Cyt average (- background)
RFP Nuc:Cyt ratio
Background 1
Background 2
Background 3
Background average
Nuc 1
Nuc 2
Nuc 3
Nuc average (- background)
Cyt 3
Cyt 2
Cyt 3
Cyt average (- background)
GFP Nuc:Cyt ratio

Related to Figure 6; Figure S5. Analysis of functional assay demonstrating disruption of nucleocytoplasmic transport in human iPSC-derived FTD-MAPT neurons.

(A) Nuclear pores are clustered within nuclear membrane invaginations in FTD-MAPT neurons. Nuclear pore subunit NUP98 (green) remains co-localised with laminB1 (red) within nuclear invaginations in *MAPT* P301-isogenic neurons and *MAPT* P301L, (120 DIV). Arrows indicate nuclear membrane invaginations. Scale bars = 5 µm. **(B)** Left panels, immunofluorescence of iPSC-derived neurons (*MAPT* P301-isogenic; 120DIV), showing β3-tubulin (grey), NLS:RFP (nuclear localisation signal fused to red fluorescent protein; red) and NES:GFP (nuclear export signal fused to green fluorescent protein; green), individual panels show combinations of signals described above from the same field. Scale bar = 10 µm. To establish the efficiency of nucleocytoplasmic transport in FTD-MAPT neurons, we calculated the ratios of NLS:RFP and NES:GFP inside nucleus and within the cytoplasm. Background (Blue), Nuclear (Nuc; yellow) and cytosolic (as determined by β3-tubulin signal; Cyt; orange) boxed areas were randomly selected and intensity of signal quantified. After background subtraction, intensity values for the nucleus and cytosol were averaged and a ratio established. Four different experiments were performed on each genotype (*MAPT* IVS10+16-A/B, *MAPT* P301L, non-demented control and MAPT P301-isogenic control) with at least six neurons per genotype.
