# Supplementary material for: Microtubules Deform the Nuclear Membrane and Disrupt Nucleocytoplasmic Transport in Tau-Mediated Frontotemporal Dementia
Source: Cell Rep. 2019 Jan 15;26(3):582–593.e5. doi: 10.1016/j.celrep.2018.12.085 (PMC6335264; doi:10.1016/j.celrep.2018.12.085)
Supplement: Document S2. Article plus Supplemental Information [file mmc5.pdf]

Cell Reports

Article

Microtubules Deform the Nuclear Membrane and Disrupt Nucleocytoplasmic Transport in Tau-Mediated Frontotemporal Dementia

Graphical Abstract

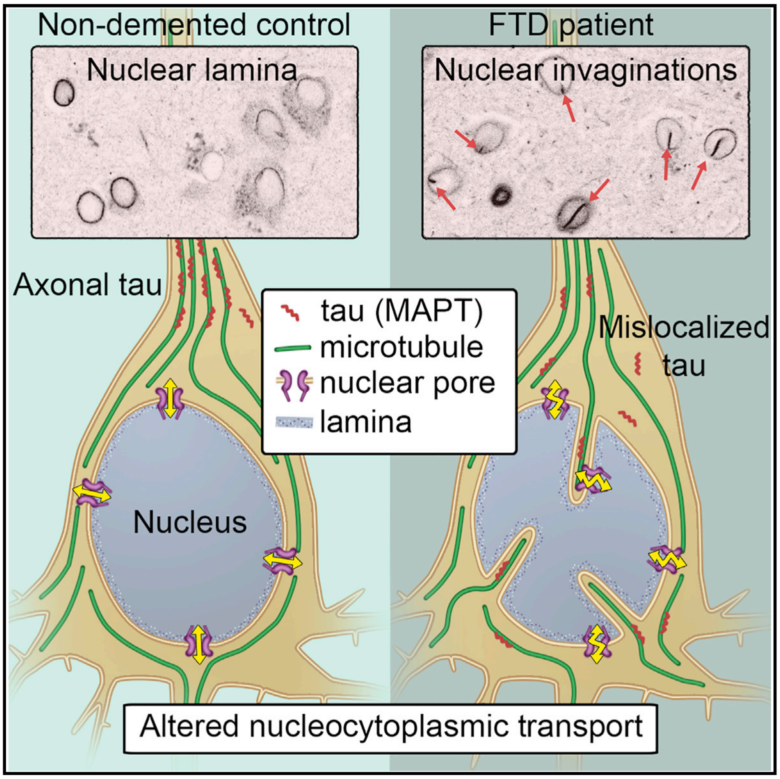

Highlights

- Tau mutations cause microtubule-mediated deformation of the nucleus in dementia
- Nuclear deformation results in defective nucleocytoplasmic transport
- Neuronal nuclei are deformed in the post-mortem frontotemporal dementia brain
- Disrupted nucleocytoplasmic transport is shared in multiple dementias

Authors

Francesco Paonessa, Lewis D. Evans, Ravi Solanki, ..., John Hardy, Stephen P. Jackson, Frederick J. Livesey

Correspondence

r.livesey@ucl.ac.uk

In Brief

Paonessa et al. show that mutations in the gene encoding the microtubule-associated protein tau that cause frontotemporal dementia result in microtubule-mediated deformation of the neuronal nucleus and disrupted nucleocytoplasmic transport. These data indicate that perturbed nucleocytoplasmic transport is a pathogenic mechanism in multiple forms of neurodegenerative disease.

Paonessa et al., 2019, Cell Reports *26*, 582–593
January 15, 2019 © 2018
https://doi.org/10.1016/j.celrep.2018.12.085

Check for updates

CellPress

OPEN ACCESS
CellPress

Cell Reports
Article

Microtubules Deform the Nuclear Membrane and Disrupt Nucleocytoplasmic Transport in Tau-Mediated Frontotemporal Dementia

Francesco Paonessa,[1,2] Lewis D. Evans,[1,2] Ravi Solanki,[1,2] Delphine Larrieu,[1,5] Selina Wray,[3] John Hardy,[3] Stephen P. Jackson,[1] and Frederick J. Livesey[1,2,4,6,*]
[1]Gurdon Institute, University of Cambridge, Tennis Court Road, Cambridge, CB2 1QN, UK
[2]Alzheimer's Research UK Stem Cell Research Centre, University of Cambridge, CB2 1QN, UK
[3]Department of Molecular Neuroscience, UCL Institute of Neurology, Queen Square, London, WC1N 3BG, UK
[4]UCL Great Ormond Street Institute of Child Health, 30 Guilford Street, London, WC1N 1EH, UK
[5]Present address: Cambridge Institute for Medical Research, University of Cambridge, Hills Road, Cambridge, CB2 0XY, UK
[6]Lead Contact
*Correspondence: r.livesey@ucl.ac.uk
https://doi.org/10.1016/j.celrep.2018.12.085

SUMMARY

The neuronal microtubule-associated protein tau, MAPT, is central to the pathogenesis of many dementias. Autosomal-dominant mutations in *MAPT* cause inherited frontotemporal dementia (FTD), but the underlying pathogenic mechanisms are unclear. Using human stem cell models of FTD due to *MAPT* mutations, we find that tau becomes hyperphosphorylated and mislocalizes to cell bodies and dendrites in cortical neurons, recapitulating a key early event in FTD. Mislocalized tau in the cell body leads to abnormal microtubule movements in FTD-MAPT neurons that grossly deform the nuclear membrane. This results in defective nucleocytoplasmic transport, which is corrected by microtubule depolymerization. Neurons in the post-mortem human FTD-MAPT cortex have a high incidence of nuclear invaginations, indicating that tau-mediated nuclear membrane dysfunction is an important pathogenic process in FTD. Defects in nucleocytoplasmic transport in FTD point to important commonalities in the pathogenic mechanisms of tau-mediated dementias and ALS-FTD due to *TDP-43* and *C9orf72* mutations.

INTRODUCTION

The microtubule-associated protein tau (MAPT; tau) is involved in the pathogenesis of several different forms of dementia, including Alzheimer's disease (AD), progressive supranuclear palsy, Pick's disease, corticobasal degeneration, and frontotemporal dementia (FTD) (Lee et al., 2001; Spillantini and Goedert, 2013). FTD is the third most common cause of dementia, after AD and vascular dementia (Rossor et al., 2010). Autosomal-dominant missense and splicing mutations in *MAPT* are causes of inherited or familial FTD (FTD-MAPT) (D'Souza et al., 1999; Goedert et al., 2012; Hutton et al., 1998). However, although it is well established that these mutations lead to hyperphosphorylation and aggregation of tau protein *in vivo* (Ballatore et al., 2007; Goedert et al., 2012), the cell biology of neuronal dysfunction and progressive neurodegeneration in this condition are currently not fully understood.

In healthy neurons, tau protein is almost exclusively localized to the axon, and several mechanisms have been suggested for its highly polarized cellular localization, including selective mRNA and protein transport, local translation, and local degradation (Wang and Mandelkow, 2016). Mislocalization and aggregation of tau in neuronal cell bodies are common features of tau-mediated dementias, including FTD and AD (Fu et al., 2016; Thies and Mandelkow, 2007; Zempel and Mandelkow, 2015). Protein aggregation is widely considered as inherently pathogenic in neurodegeneration (Fitzpatrick et al., 2017; Hernández-Vega et al., 2017), altering many cellular functions, most notably autophagy and proteostasis (Bence et al., 2001; Caballero et al., 2018; Lim and Yue, 2015). However, how *MAPT* mutations lead to tau hyperphosphorylation and mislocalization, the effects of this mislocalization on neuronal cell biology, and how this contributes to neuronal dysfunction and neurodegeneration all remain poorly understood.

As a typical microtubule-binding protein, tau has several roles in regulating microtubule function and intracellular transport (Wang and Mandelkow, 2016). Tau binds both alpha and beta tubulin subunits of microtubules and has been demonstrated to both stabilize and promote microtubule growth (Kadavath et al., 2015; Witman et al., 1976). The presence of tau on microtubules can alter directions and rates of axonal transport (Dixit et al., 2008; Trinczek et al., 1999). Tau is a natively disordered protein and has recently been found to undergo fluid phase transitions at higher concentrations, nucleating microtubules when it does so (Hernández-Vega et al., 2017). Therefore, it is likely that the changes in tau levels, post-translational modifications, and cellular localization that occur in dementia lead to alterations in microtubule biology, particularly in the neuronal cell body.

To address the question of how *MAPT* mutations lead to neuronal dysfunction and neurodegeneration, we investigated the effects of two different classes of *MAPT* mutations on the cell biology of human iPSC-derived cortical neurons. We find that both missense and splicing *MAPT* mutations cause mislocalization of tau to the cell bodies of neurons and marked changes

This is an open access article under the CC BY-NC-ND license (http://creativecommons.org/licenses/by-nc-nd/4.0/).

in microtubule dynamics. Microtubules in the cell bodies of FTD-MAPT neurons actively deform the nuclear membrane, disrupting nucleocytoplasmic transport. Defects in nuclear envelope function, including nucleocytoplasmic transport, are an important pathological process in ALS-FTD because of repeat expansions in *C9orf72* and *TDP-43* mutations (Chou et al., 2018; Zhang et al., 2015, 2016, 2018). Our findings demonstrate that dysfunction of the nuclear membrane due to altered microtubule dynamics is a pathogenic process in dementias involving tau, expanding the group of neurodegenerative diseases that involve disrupted nucleocytoplasmic transport and suggesting common mechanisms of neuronal dysfunctional in these heterogeneous conditions.

RESULTS

Increased Phosphorylation and Altered Cellular Localization of Tau in FTD-MAPT Neurons

To study the effects on neuronal cell biology of FTD-MAPT mutations, we generated excitatory cortical neurons (Shi et al., 2012b) from induced pluripotent stem cells (iPSCs) derived from individuals with different autosomal-dominant mutations in *MAPT* that are causal for early-onset FTD (Figures 1 and S1). We studied two different types of mutations: the *MAPT* IVS10+16 autosomal-dominant mutation, which increases inclusion of exon 10, encoding the second microtubule-binding repeat and thus altering the ratio of three (3R) and four (4R) tau isoforms (Hutton et al., 1998; Sposito et al., 2015), and the autosomal-dominant *MAPT* P301L missense mutation that produces an aggregation-prone form of tau (Wang and Mandelkow, 2016) (Figures 1 and S1). The cortical identity of the neurons generated and the reproducibility of the culture compositions among genotypes was confirmed by assessing the expression of a set of classifier genes that define different neuronal cell types (Figure S1).

Total tau content was similar in neurons of each genotype, collectively referred to here as FTD-MAPT neurons (Figure 1A). Notably, tau phosphorylation was increased in FTD-MAPT neurons compared with controls (Figure 1A), including at Ser404 and Ser202/Thr205 (AT8), epitopes typically hyperphosphorylated in tau-mediated dementias (Alonso et al., 2004; Wang et al., 2013). As both mutations are dependent on expression of exon 10 of *MAPT*, we confirmed translation of exon 10 in neurons generated from all iPSC lines by western blotting and mass spectrometry (Figures 1B, 1C, and S1). No significant difference in insoluble, aggregated tau was detected by sarkosyl extraction between neurons of each genotype (Figure S1).

Mislocalization of tau from axons to neuronal cell bodies and dendrites is an early event in FTD *in vivo* (Götz et al., 1995; Hoover et al., 2010; Kowall and Kosik, 1987). As expected, control neurons showed a predominantly axonal distribution of tau, with tau largely absent from MAP2-positive neuronal cell bodies and dendrites (Figure 1D). In contrast, tau was commonly present in MAP2-positive cell bodies and dendrites in both *MAPT* IVS10+16 and P301L neurons (Figures 1D and 1E). Furthermore, tau within cell bodies and dendrites of FTD-MAPT neurons was hyperphosphorylated, as detected by AT8-immunoreactivity (phospho-S202/T205) (Figure 1D).

Microtubules Invade the Nucleus in FTD-MAPT Neurons

Given the mislocalization of tau to the cell bodies of FTD-MAPT neurons, we studied neuronal microtubule dynamics in control and FTD-MAPT neurons (Figures 2 and S2). Actively extending microtubules were live-imaged in iPSC-derived neurons of each genotype by expression of GFP-tagged EB3 (Figure 2; Videos S1 and S2), the microtubule plus-end binding protein (+TIP) (Akhmanova and Steinmetz, 2008). Total microtubule movements were not different between non-demented control and FTD-MAPT neurons, with similar rates of extensions and retractions measured among the various genotypes (Table S1).

However, microtubule dynamics were qualitatively different in the cell bodies of control neurons compared with FTD-MAPT neurons. Non-demented control neurons typically had many actively growing microtubules within the cell body that extended around a smooth, oval nucleus (Figures 2A and 2B; Video S1). In contrast, many FTD-MAPT neurons had microtubules with plus ends projecting into the nucleus (15 of 26 FTD-MAPT neurons) (Figure 2B), an event that was infrequently detected in both groups of control neurons (2 of 19) (Figure 2B). Notably, those microtubules that abnormally projected into the nucleus in FTD-MAPT neurons frequently originated from a pronounced focus that resembled a microtubule organizing center (Figure 2A; Video S1). These pronounced foci were not detected in either of the control neuronal lines. We confirmed that the EB3+ microtubules that project into the neuronal nucleus are dynamically growing microtubules, as the mobility of EB3+ comets was greatly reduced following acute microtubule depolymerization (with nocodazole) (Figures S2Band S2C; Video S2).

Microtubules Deform the Nuclear Envelope in FTD-MAPT Neurons

Microtubules couple to the nuclear membrane through the LINC complex (Crisp et al., 2006). This physical association results in transmission of mechanical forces that influence nuclear shape and integrity (Chang et al., 2015), affecting the function of the nuclear envelope (Webster et al., 2009). Given the abnormal projection of microtubules into the nucleus in FTD-MAPT neurons, we studied the shape of the nuclear envelope in iPSC-derived neurons. Marked differences were present in nuclear shape between non-demented controls and FTD-MAPT neurons, as demonstrated by large folds, or invaginations, of the laminB1-positive inner nuclear lamina within the nucleus (Figure 3). The neuronal identity of invaginated cells was confirmed by the co-staining with the cortical deep layer transcription factor CTIP2 and the pan-neuronal protein β3-tubulin (Figure 3).

Quantification of the proportions of neurons with deformation of the nuclear membrane, as defined by the presence of laminB1-positive regions within the nucleus, (Figures 3B and S3), demonstrated that deep nuclear invaginations were present in approximately 25% of *MAPT* P301L and 40% of *MAPT* IVS10+16 neurons, compared with fewer than 10% of control neurons (Figure 3B). To confirm that microtubules actively deformed the nucleus in FTD-MAPT neurons, we acutely depolymerized microtubule with the small molecule nocodazole. This

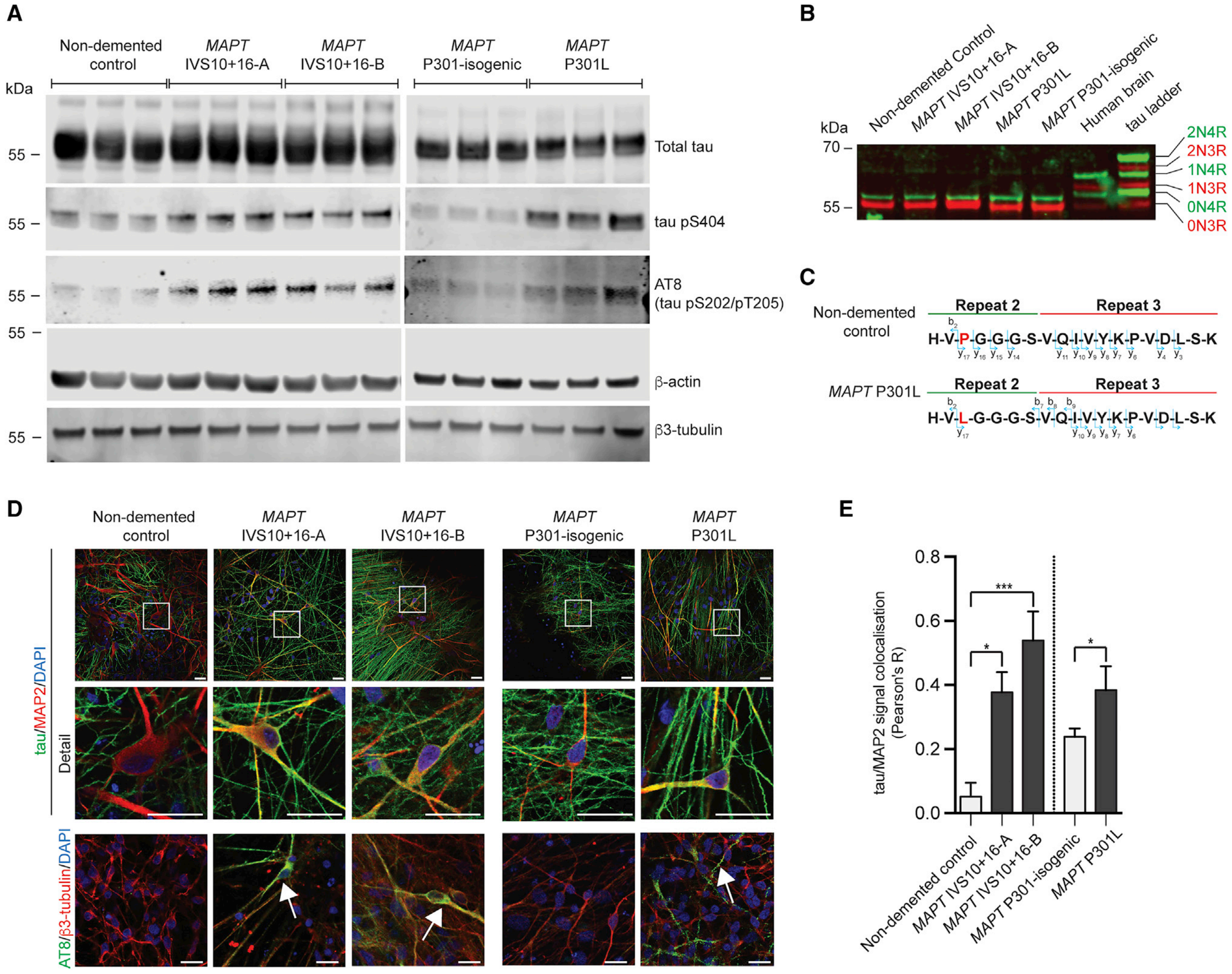

Figure 1. Increased Phosphorylation and Altered Cellular Localization of Tau in FTD-MAPT Neurons

(A) Phosphorylated tau (pS404; AT8 [pS202/pT205]) is increased as a fraction of total tau (epitope 243–441) in frontotemporal dementia (FTD)-MAPT neurons (*MAPT* IVS10+16-A/B and *MAPT* P301L) compared with non-demented and MAPT P301 isogenic control neurons (induced pluripotent stem cell [iPSC]-derived neurons at 120 days *in vitro* [DIV]; three biological replicates). β-actin and β3-tubulin were used as controls. Molecular weight (kDa) is indicated.

(B) Tau isoforms with three (3R; red) or four (4R; inclusion of region 2; green) microtubule-binding regions were detected by western blot analysis of dephosphorylated protein extracts from iPSC-derived control and FTD-MAPT cortical neurons (120 DIV) and from post-mortem human cerebral cortex (non-demented individual). Tau isoforms were identified relative to a commercial tau ladder (Sigma). Molecular weight (kDa) is indicated.

(C) Peptide sequences identified by tau immunoprecipitation (IP)/mass spectrometry from iPSC-derived cortical neurons, confirming the inclusion of repeat 2 (corresponding to exon 10) of 4R tau. In *MAPT* P301L neurons, both proline and leucine were identified at position 301 (highlighted red). See also Figure S1.

(D) Tau protein is mislocalized to MAP2-positive cell bodies and dendrites in iPSC-derived FTD-MAPT neurons. Confocal images of iPSC-derived control and FTD-MAPT neurons (120 DIV; tau, green; MAP2, red; DAPI, blue). Hyperphosphorylated, AT8-positive tau (AT8; green) is found in cell bodies of FTD-MAPT neurons (arrows) but not in controls (β3-tubulin, red; DAPI, blue). Scale bars, 20 μm.

(E) Increased co-localization of tau and MAP2 protein in FTD-MAPT neurons, compared with non-demented control neurons, analyzed by Pearson's R correlation (control lines, gray bars; FTD-MAPT lines, black).

Significance was determined for three-sample comparison of non-demented control and two *MAPT* IVS10+16 lines using one-way ANOVA followed by Tukey's test (*$p < 0.05$ and ***$p < 0.001$). Pairwise comparison of the *MAPT* P301L line and its isogenic control was carried out using Student's t test (*$p < 0.05$); error bar represents SEM; n = 3 independent experiments.

See also Figure S1.

significantly reduced the proportion of neurons with nuclear invaginations and restored round nuclear morphology (Figures 3C and 3D). We conclude that the pronounced deformations of the neuronal nuclear membrane in FTD-MAPT neurons are actively mediated by microtubules.

Super-resolution Imaging Demonstrates Close Apposition of Tau and Tubulin within Nuclear Lamina Invaginations

To further study the spatial relationships between tau, microtubules, and the nuclear envelope, we conducted a detailed

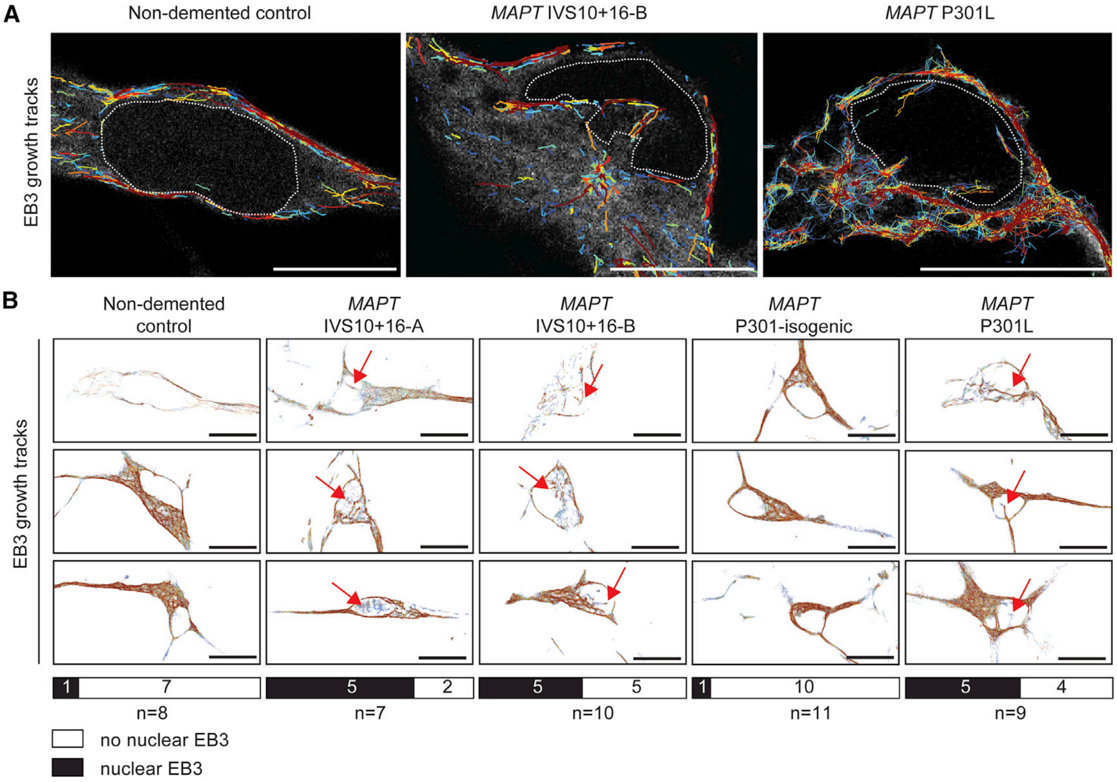

Figure 2. Microtubules Invade the Nucleus in FTD-MAPT Neurons

(A) Total microtubule trajectories (cumulative over a 200 s interval) overlaid on stills from GFP-EB3 live imaging (gray) demonstrate multiple microtubule extensions into the nuclei of FTD-MAPT neurons (*MAPT* IVS10+16-B and *MAPT* P301L; 120 DIV), compared with non-demented control neurons; white dotted line indicates the nuclear membrane.

(B) GFP-EB3 growth tracks from representative neurons (cumulative over a 200 s interval) from FTD-MAPT neurons (*MAPT* IVS10+16-A/B and *MAPT* P301L) compared with non-demented and *MAPT* P301 isogenic control neurons (iPSC-derived neurons at 120 DIV; three biological replicates).

Red arrows indicate examples of trajectories within the nucleus. Bars indicate the number of sampled neurons with (black), and without (white), nuclear EB3 growth tracks; n = number of imaged neurons. Scale bars, 10 μm.

See also Video S1, Table S1, and Figure S2.

analysis of the neuronal nucleus in iPSC-derived neurons using three-dimensional (3D) stimulated emission depletion (STED) super-resolution imaging. Three-dimensional STED imaging demonstrated that invaginations of the nuclear lamina present in FTD-MAPT neurons commonly extended deeply into the nucleus, in some cases traversing the entire length of the nucleus, forming pronounced folds (Figure 4A). In comparison, nuclei from non-demented controls had a regular, smooth morphology, with few examples of nuclear lamina invaginations (Figure 4A).

As observed by confocal microscopy, tau protein was found to be abundant in the cell bodies of FTD-MAPT neurons by STED imaging and in those neurons was closely apposed to the outer nuclear membrane (Figure 4B). Both tau protein and neuronal tubulin were found within nuclear lamina invaginations in FTD-MAPT neurons (Figure 4B). STED imaging demonstrated that tau within nuclear membrane invaginations is within hundreds of nanometers of the nuclear lamina (Figure 4C). Given that laminB1 filaments line the inner surface of the nuclear envelope, which is typically of the order of 15–60 nm in width (Burke and Stewart, 2013; Gerace and Huber, 2012), we conclude that tau is in close proximity to proteins in the outer membrane of the nuclear envelope in FTD-MAPT neurons.

Tau-Containing Nuclear Lamina Invaginations in Neurons of the Post-mortem FTD-MAPT Cerebral Cortex

Having identified nuclear lamina defects in iPSC-derived FTD-MAPT neurons, we asked whether alterations of the nuclear lamina are also a feature of FTD-MAPT *in vivo*. To do so, we studied the incidence of invaginations of the nuclear lamina in the frontal and temporal cortex from two separate cohorts from independent brain banks, both containing individuals diagnosed with FTD due to *MAPT* IVS10+16 mutations and compared with age-matched non-demented controls. These cohorts were analyzed separately using different methods for detecting laminB1. We quantified the fraction of all nuclei with invaginations within each brain region (Figure 5). Similar results were obtained in both cohorts: the frequency of nuclear lamina invaginations was higher in the deep cortical layers in post-mortem cerebral cortices from individuals with FTD due to the *MAPT*

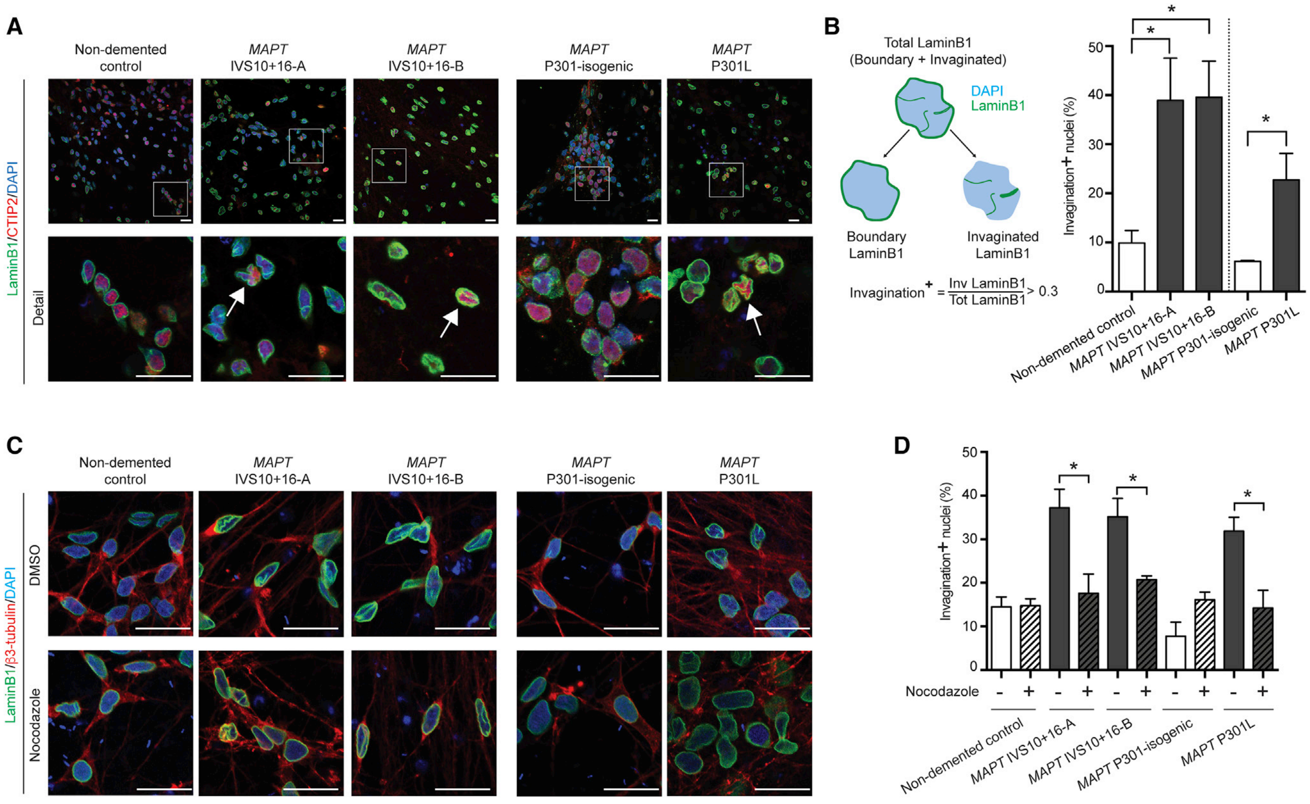

Figure 3. Microtubules Deform the Nuclear Envelope in FTD-MAPT Neurons

(A) Marked abnormalities of nuclear lamina shape in FTD-MAPT neurons. Confocal images of the nuclear lamina (laminB1, green) in FTD-MAPT neurons (*MAPT* IVS10+16-A/B and *MAPT* P301L; neuronal transcription factor CTIP2, red) compared with non-demented and *MAPT* P301 isogenic control neurons (120 DIV). White arrows indicate examples of nuclei with pronounced deformation of the nuclear lamina.

(B) FTD-MAPT neurons have increased numbers of cells with deformed nuclear membranes, as defined by the shape of the inner nuclear lamina. Schematic of image analysis method used to quantify nuclear invaginations, as a measure of distortion of the nuclear membrane: nuclear area was established using DAPI (blue), and nuclear lamina (laminB1, green) was assigned as either nuclear boundary or invaginated (i.e., within the nucleus). The fraction of total laminB1 that was invaginated was used empirically to define a threshold for defining neurons as having nuclear membrane invaginations (see Figure S3 for details). Between 25% (*MAPT* P301L) and 40% (*MAPT* IVS10+16) of FTD-MAPT neurons have nuclear invaginations, compared with fewer than 10% of control neurons. Significance was determined for non-demented control and two *MAPT* IVS10+16 lines using one-way ANOVA followed by Tukey's test (*$p < 0.05$); pairwise comparison of the *MAPT* P301L line and its isogenic control was carried out using Student's t test (*$p < 0.05$); error bar represents SEM; n = 3 independent experiments.

(C) Acute depolymerization of microtubules reverses nuclear lamina invaginations and restores rounded nuclear shapes. Confocal images of control and FTD-MAPT neurons (using genotypes described in A; 120 DIV), treated with DMSO (vehicle) or 10 μM nocodazole for 3 h (laminB1, green; β3-tubulin, red; DAPI, blue). Scale bars, 10 μm.

(D) The proportion of FTD-MAPT neurons with nuclear lamina invaginations is significantly reduced by nocodazole treatment.

Quantification of neurons with abnormalities of the nuclear lamina was carried out as in B. n = 3 independent experiments; error bars represent SEM. Significance was determined using one-way ANOVA followed by Tukey's test (*$p < 0.05$); error bar represents SEM; n = 3 independent experiments.

See also Figure S3.

IVS10+16 mutation, compared with non-demented control individuals (Figures 5 and S4). This was the case in both frontal and temporal cortex (Figures 5B, 5E, and 5F).

Data from iPSC-derived FTD-MAPT neurons suggested that laminB1-positive nuclear invaginations would be associated with the presence of phosphorylated tau within the neuronal cell body. Consistent with this, we found that the nuclear lamina was grossly disrupted in neurons that had high levels of hyperphosphorylated (AT8+) tau and neurofibrillary tangles in the post-mortem FTD-MAPT IVS10+16 cerebral cortex (Figures 5C and S4B), and those neurons frequently contained pronounced nuclear lamina invaginations (Figures 5C and S4B). Furthermore, nuclear lamina invaginations in such neurons also commonly contained AT8-positive hyperphosphorylated tau (Figure 5C).

Disrupted Nucleocytoplasmic Transport in FTD-MAPT Neurons

Abnormalities of the nuclear lamina are also found in aging diseases, such as Hutchinson-Gilford progeria syndrome (Broers et al., 2006). Nuclear membrane distortion in response to mechanical forces leads to deleterious effects on many aspects of nuclear function, disrupting nucleocytoplasmic transport (Kelley et al., 2011). We confirmed that the nuclear lamina/membrane invaginations present in iPSC-derived FTD-MAPT neurons also

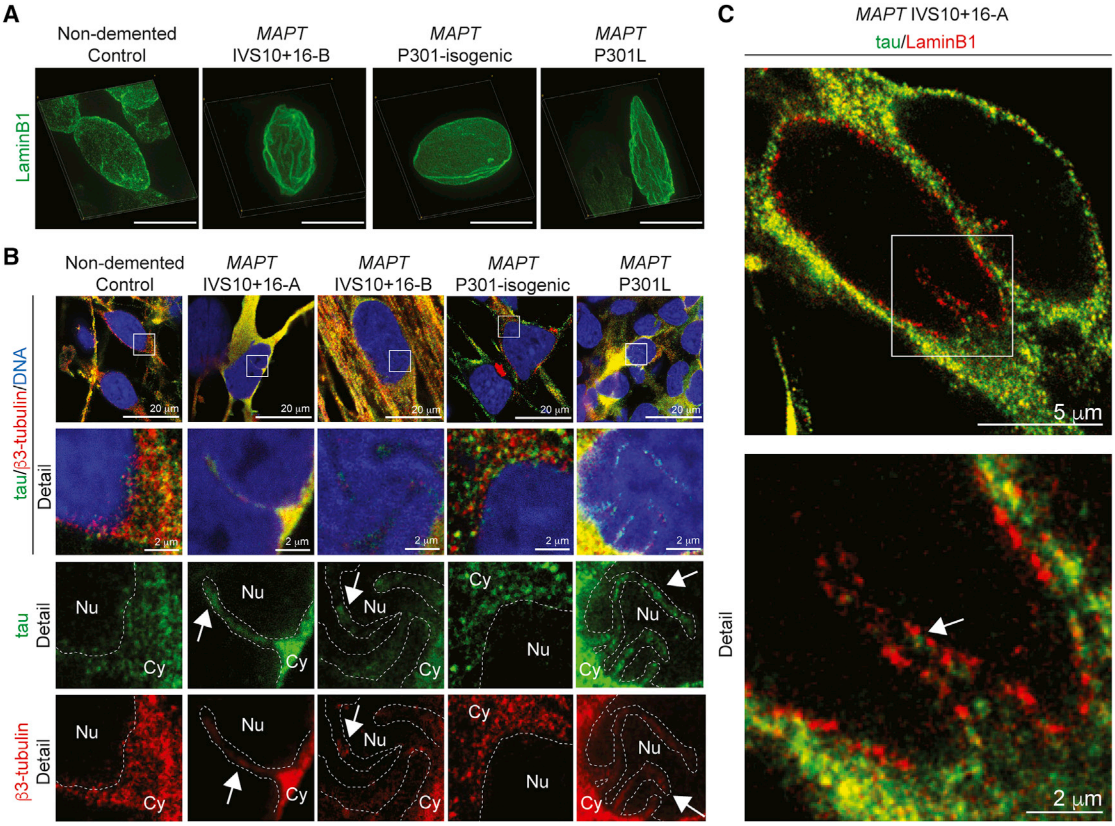

Figure 4. Super-resolution Imaging Demonstrates Close Apposition of Tau and Tubulin within Nuclear Lamina Invaginations

(A) Three-dimensional reconstructions using STED imaging of the nuclear lamina (laminB1, green) in FTD-MAPT iPSC-derived neurons (*MAPT* IVS10+16 and *MAPT* P301L) reveal pronounced nuclear invaginations compared with non-demented and MAPT P301-isogenic control neurons (120 DIV). Scale bar, 10 μm.
(B) Tau is in close proximity to the nuclear lamina within nuclear invaginations of FTD-MAPT neurons. STED imaging of control and FTD-MAPT iPSC-derived neurons (using genotypes described in A; 120 DIV; tau, green; β3-tubulin, red; DNA, Yo-Pro, blue). Detail from white boxes in upper panels, showing both merge of all channels, and single channel images of tau (green) or β3-tubulin (red). Dashed lines indicate the boundary between the nucleus (Nu) and cytoplasm (Cy). Arrows indicate invaginations into the nucleus.
(C) Nuclear invaginations are lined with nuclear lamina and contain tau. STED imaging of *MAPT* IVS10+16-A neurons (120 DIV; tau, green; laminB1, red). Arrow indicates tau within a nuclear invagination, in close proximity to the laminB1-positive inner nuclear lamina.

contained nuclear pores within these membrane folds, with nuclear pores (labeled by NUP98) co-localizing with laminB1-positive invaginations (Figure S5A).

To assess whether alterations in the nuclear membrane in FTD-MAPT neurons result in defects in nucleocytoplasmic transport, we expressed NES:GFP and NLS:RFP from a single construct in iPSC-derived neurons (Mertens et al., 2015). This assay enables measurement of the integrity of both nuclear localization and accumulation and cytoplasmic retention and nuclear exclusion within individual neurons (Figures 6A and S5B). Control iPSC-derived neurons had discrete cellular distributions of each protein, with prominent nuclear RFP and cytosolic GFP (Figure 6B). In contrast, localization of NLS:RFP was altered in FTD-MAPT neurons such that there was a marked decrease in the nuclear/cytoplasmic RFP ratio (Figure 6C). Conversely, nuclear exclusion of NES:GFP was reduced in FTD-MAPT neurons, with an increase of GFP within the nucleus (Figure 6C). Together, these data demonstrate defects in the selective permeability of the nuclear envelope in FTD-MAPT neurons, indicating a general failure of nucleocytoplasmic transport within FTD-MAPT neurons.

Defective nucleocytoplasmic transport may be a cumulative phenomenon due to protracted nuclear membrane damage, or an acute process due to microtubule-mediated nuclear membrane deformation. To distinguish between these mechanisms, we acutely depolymerized microtubules with nocodazole in control and FTD-MAPT neurons and quantified nucleocytoplasmic transport using the NES:GFP/NLS:RFP assay. This treatment corrected the distribution of both NES:GFP and NLS:RFP in FTD-MAPT neurons, restoring the nucleocytoplasmic ratios to the level of healthy control neuron (Figure 6D). We conclude that defective nucleocytoplasmic transport in FTD-MAPT neurons is an ongoing process due to microtubule-mediated deformation of the nuclear membrane.

DISCUSSION

The cellular and molecular biology of the pathogenesis of FTD due to *MAPT* mutations is not well understood. Currently, it is thought that *MAPT* mutations all lead to tau protein aggregation and that protein aggregation is the primary driver

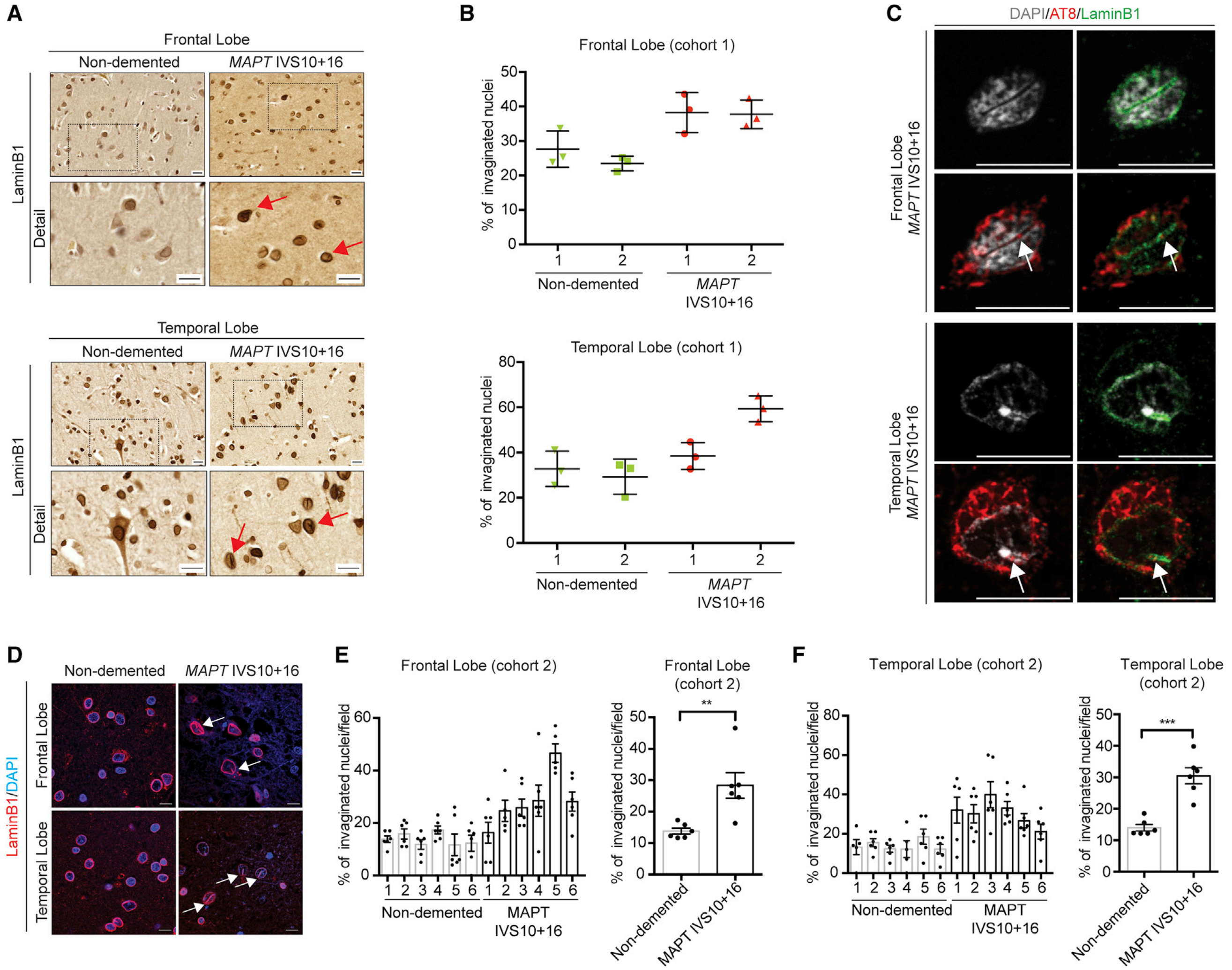

Figure 5. Tau-Containing Nuclear Lamina Invaginations in Neurons of the Post-mortem FTD-MAPT Cerebral Cortex

(A) Increased incidence of laminB1-positive nuclear invaginations *in vivo*, in post-mortem FTD-MAPT IVS10+16 cortex compared with age-matched controls (cohort 1). Immunohistochemistry of laminB1 in (top) frontal and (bottom) temporal cortex from individuals with FTD due to the *MAPT* IVS10+16 mutation or age-matched controls (non-demented). Red arrows indicate nuclei exhibiting nuclear invaginations.

(B) Percentage of invaginated nuclei in deep layers of frontal and temporal cortex of two control (green) and two *MAPT* IVS10+16 individuals (red), calculated from 20 individual imaging fields (cohort 1). Nuclei were scored by three observers, blinded to the identity of the post-mortem samples, and the averages of the three measurements are shown. Error represents SEM.

(C) Hyperphosphorylated, AT8+ tau within nuclear lamina invaginations in neurons of the frontal (top) and temporal (bottom) cortex of an individual carrying *MAPT* IVS10+16 mutation. Representative neurons, showing an extensive nuclear invagination (laminB1, green; DAPI, gray) containing hyperphosphorylated tau (AT8, red). White arrows indicate nuclear invaginations. Scale bars, 10 μm. See also Figure S4.

(D) Representative confocal images from post-mortem FTD-MAPT IVS10+16 cortex compared with age-matched controls (cohort 2) showing laminB1 (red) and DAPI (blue); white arrows indicate nuclear invaginations. Scale bar, 10 μm.

(E and F) Left: percentage of invaginated nuclei in deep layers of frontal (E) and temporal (F) cortex from six aged-matched controls and six FTD-MAPT IVS10+16 individuals (cohort 2) quantified using the analysis method described in Figures 3 and S3. Points indicate quantifications from individual fields. Right: pairwise comparison of the control and FTD-MATP groups show increased percentage of invaginated nuclei in pathology (points indicate individuals; cohort 2) carried out using Student's t test (***$p < 0.001$); error bar represents SEM.

of neurodegeneration (Ballatore et al., 2007; Spillantini and Goedert, 2013). However, protein aggregation may represent only the late stage of the disease, and the processes preceding and leading to neurofibrillary tangle formation and cellular dysfunction remain to be elucidated. Here we report the use of human stem cell systems to study the effects of those mutations on neuronal cell biology, finding that tau-mediated dementias are accompanied by defective neuronal nucleocytoplasmic transport.

Focusing on two different types of *MAPT* mutations causal for FTD, we have found that both IVS10+16 and P301L mutations lead to marked defects in nucleocytoplasmic transport in human

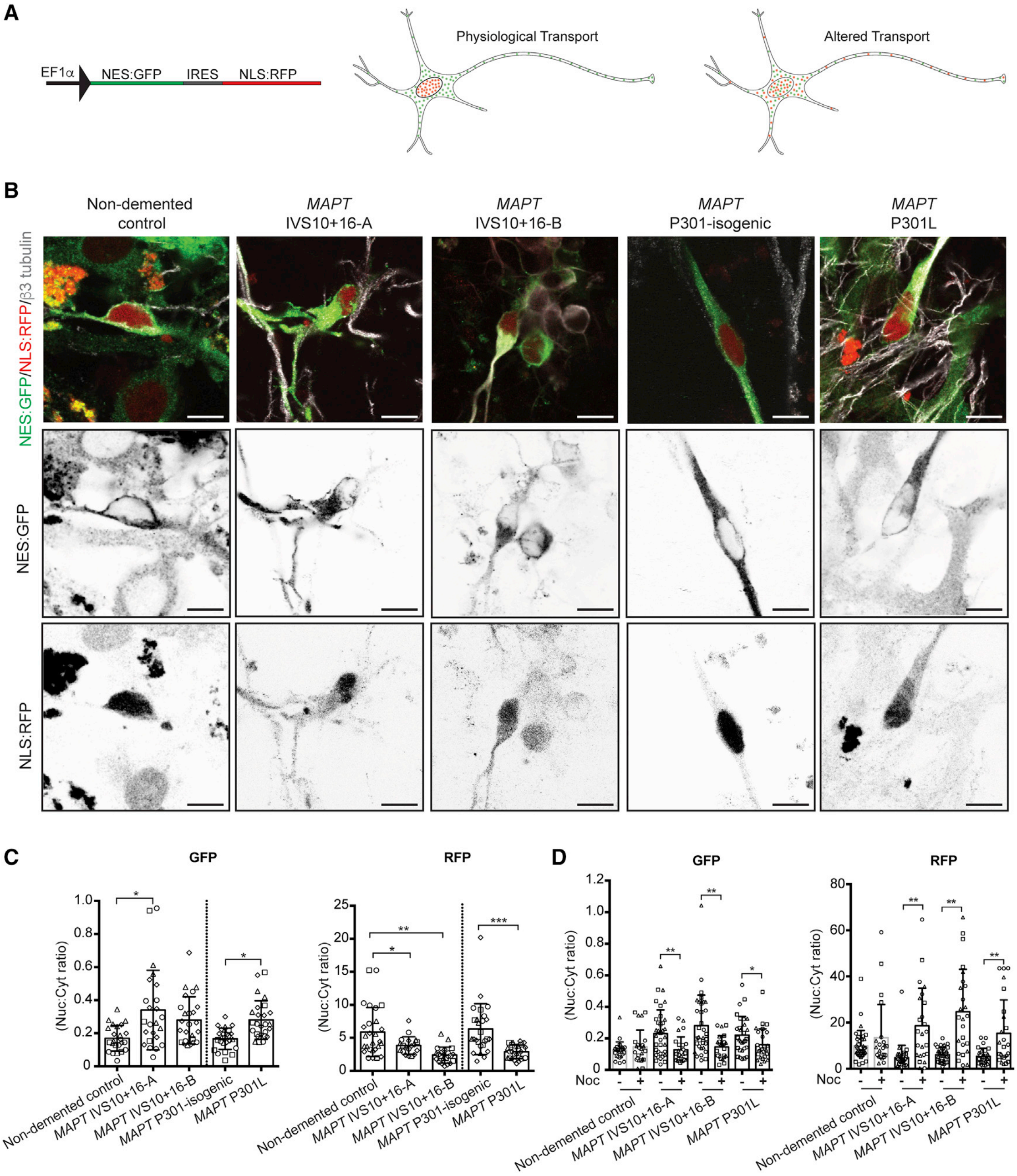

Figure 6. Disrupted Nucleocytoplasmic Transport in FTD-MAPT Neurons

(A) Functional assay demonstrates disrupted nucleocytoplasmic transport in human iPSC-derived FTD-MAPT neurons. Schematic illustrates lentiviral vector for co-expression of NES:GFP (nuclear export signal fused to GFP) and NLS:RFP (nuclear localization signal fused to RFP) within human neurons and relative distributions of both proteins in healthy neurons and in cells with defective nucleocytoplasmic transport.

(B) Representative confocal images of control and FTD-MAPT neurons (*MAPT* IVS10+16-A/B and *MAPT* P301L; all 120 DIV) expressing GFP:NES and RFP:NLS (GFP, green; RFP, red; β3-tubulin). Grayscale images of NES-GFP and NLS-RFP localization in representative cells of each genotype are shown: FTD-MAPT neurons show an increase of GFP within the nucleus and a reduction in nuclear localization of NLS-RFP. Scale bars, 20 μm.

(C) Quantification of the nuclear/cytoplasmic ratio for both NES:GFP and NLS:RFP demonstrates altered nuclear transport in FTD-MAPT genotypes relative to controls: NES:GFP is increased in the nuclei of FTD-MAPT neurons, whereas NLS:RFP is decreased (points indicate quantifications from individual fields, different shapes indicate measurements from different experiments). Significance was determined for non-demented control and two *MAPT* IVS10+16 lines using one-way ANOVA followed by Dunnett's test (*$p < 0.05$, **$p < 0.01$, and ***$p < 0.001$). Pairwise comparison of the *MAPT* P301L line and its isogenic control was performed using Student's t test (*$p < 0.05$); error bar represents SEM; n = 4 independent experiments.

(legend continued on next page)

neurons. We find that both missense and splicing mutations in *MAPT* alter tau protein localization and phosphorylation within iPSC-derived neurons within 4 months in cell culture, recapitulating a well-described aspect of early FTD pathology *in vivo* (Götz et al., 1995; Hoover et al., 2010; Kowall and Kosik, 1987), without detectable tau aggregation. Mislocalization of tau in the cell bodies of FTD-MAPT neurons in culture leads to marked changes in microtubule dynamics, causing deformation of the nuclear membrane both in cell culture and in the human FTD-MAPT cortex *in vivo*. Disruption of the nuclear lamina is commonly associated with dysfunction of the nuclear envelope, and we find marked disruption of nucleocytoplasmic transport in FTD-MAPT neurons. Disrupted nucleocytoplasmic transport is due to ongoing microtubule-mediated nuclear membrane deformation, as it is corrected by acute microtubule depolymerization. Together, these data indicate that perturbation of the function of the nuclear membrane and disruption of nucleocytoplasmic transport is an important pathological process in FTD due to *MAPT* mutations.

An early event in FTD is the mislocalization of tau from axons to cell bodies and dendrites, and this key stage in disease progression is also an early event in iPSC-derived models of FTD-MAPT. *In vivo*, mislocalization of tau is typically associated with tau hyperphosphorylation (Götz et al., 1995; Spillantini and Goedert, 2013). We find this also occurs in iPSC-derived FTD-MAPT neurons, where we detected increased tau phosphorylation at serine 202/threonine 205 (the AT8 epitope) and also at serine 404. The sequence in which mislocalization and hyperphosphorylation take place in FTD *in vivo*, and in iPSC-derived FTD-MAPT neurons in culture, is not currently clear, nor are the mechanisms by which these processes occur. The appearance of tau within cell bodies and dendrites indicates a breakdown of the cellular polarity mechanisms that maintain the axonal enrichment of tau protein and its exclusion from the somatodendritic compartment, mechanisms that are poorly understood.

The two heterozygous, dominant *MAPT* mutations studied here have different effects on tau protein in neurons. The *MAPT* P301L missense mutation, like many missense mutations in the microtubule-binding region domain of *MAPT*, increases the tendency of tau to aggregate in cell-free assays and in transgenic mouse models (von Bergen et al., 2005; Lewis et al., 2000; Shammas et al., 2015). In contrast, the IVS10+16 mutation is not a coding mutation but rather is an intronic single base change that favors the inclusion of exon 10 in the *MAPT* mRNA, increasing the amount of tau containing four microtubule-binding repeats (4R), relative to the three-repeat (3R) form (Hutton et al., 1998). However, despite these differences, the changes in the forms of tau in both *MAPT* P301L and IVS10+16 neurons both lead to mislocalization and increased phosphorylation of tau. This finding suggests that either the presence of a pool of P301L tau, or a shift in the 3R/4R tau ratio, alter a common pathway that regulates tau distribution within neurons, tau phosphorylation, or both.

In both *MAPT* IVS 10+16 and P301L mutant neurons, the appearance of tau in cell bodies is accompanied by marked qualitative changes in neuronal microtubule dynamics. Microtubules in FTD-MAPT neurons actively deform the nuclear envelope, which we find can be reversed by depolymerization of microtubules. Tau has multiple roles in stabilizing microtubules (Wang and Mandelkow, 2016), and microtubules are coupled to the nuclear membrane through the LINC complex (Chang et al., 2015; Crisp et al., 2006; Luo et al., 2016). Therefore, it is likely that the overall effect of the presence of tau in the cell body is to promote microtubule stability, leading to increased pushing forces on the nuclear membrane and the formation of invaginations in the nuclear membrane. As tau has recently also been found to promote microtubule nucleation when undergoing phase transitions at high concentration (Hernández-Vega et al., 2017), accumulation of tau in the cell body may also lead to increased pushing forces on the nucleus by facilitating microtubule nucleation.

Alterations in nuclear shape and nuclear membrane function are a common feature of cellular aging, including in the nervous system, and are associated with multiple deleterious changes in nuclear biology, including chromatin changes and disrupted nucleocytoplasmic transport (Frost, 2016; Oberdoerffer and Sinclair, 2007). *Drosophila* models of FTD, with neuronal expression of human *MAPT* R406W, have nuclear shape abnormalities and chromatin changes (Frost et al., 2016). Recently, nuclear membrane disruption has been reported in a *MAPT* P301L transgenic mouse model of FTD (Eftekharzadeh et al., 2018). Perturbations of the nuclear lamina have been described in the post-mortem AD brain (Frost et al., 2016), including the juxtaposition of neurofibrillary tangles of tau with the nuclear membrane (Sheffield et al., 2006). We also find here an increase in nuclear invaginations in neurons of the human post-mortem *MAPT* IVS10+16 cortex, and the presence of hyperphosphorylated tau within nuclear invaginations in tangle-bearing neurons. Together, these different studies are consistent with a pathological effect of tau within the neuronal cell body in FTD and AD, whereby the presence of tau alters microtubule biology, resulting in pronounced abnormalities of the neuronal nucleus and defective nucleocytoplasmic transport. Recent findings have shown a direct interaction of aggregated tau with the nuclear pore complex (Eftekharzadeh et al., 2018), suggesting that the microtubule disruption of nuclear pore function reported here may be mediated by tau protein at the nuclear membrane.

Microtubule deformation of the nucleus is a phenotype also seen in classic laminopathies such as the accelerated aging disorder Hutchinson-Gilford progeria syndrome (HGPS), in which the primary defect is due to mutant lamin A/C protein (Capell

(D) Effect of nocodazole (Noc) on the nuclear/cytoplasmic ratio for both NES:GFP and NLS:RFP. Nuclear/cytoplasmic ratio is restored in the presence of nocodazole (+) in FTD-MATP neurons compared with vehicle (–) treated neurons (points indicate quantifications from individual fields, different shapes indicate measurements from different experiments).

Significance was determined using one-way ANOVA followed by Dunnett's test (*$p < 0.05$, **$p < 0.01$, and ***$p < 0.001$); error bar represents SEM; n = 3 independent experiments.

See also Figure S5.

and Collins, 2006). In that case, microtubules also contribute to nuclear deformations, leading to defects in nucleocytoplasmic transport (Kelley et al., 2011; Larrieu et al., 2014; Snow et al., 2013). Changes in nuclear envelope function in other neurodegenerative diseases, including ALS-FTD due to repeat expansions in *C9orf72*, Huntington's disease, and AD, have recently been reported (Freibaum et al., 2015; Grima et al., 2017; Jovičić et al., 2015; Zhang et al., 2015).

Our finding here of disruption of the neuronal nuclear membrane as a consequence of *MAPT* mutations in FTD extends this pathogenic mechanism to dementias in which protein aggregation has been thought to be the primary driver of neurodegeneration. These data suggest that dysfunction of the nuclear membrane may be a common pathogenic process in diverse neurodegenerative diseases, which could be targeted therapeutically with agents that regulate microtubule functions, nucleocytoplasmic transport, and/or associated processes.

STAR★METHODS

Detailed methods are provided in the online version of this paper and include the following:

- KEY RESOURCES TABLE
- CONTACT FOR REAGENT AND RESOURCE SHARING
- EXPERIMENTAL MODEL AND SUBJECT DETAILS
  - Human iPSC lines
  - Human post-mortem brain sections
- METHOD DETAILS
  - Generation of iPSC-derived cortical neurons and drug treatments
  - Protein extraction and western blot analysis
  - Sarkosyl extraction
  - Immunoprecipitation and mass spectrometric analysis of intracellular tau
  - RNA extraction and qRT-PCR analysis
  - Confocal microscopy and image analysis
  - Staining of human formalin-fixed paraffin embedded (FFPE) brain sections
  - Live imaging of microtubule dynamics
  - Nucleocytoplasmic transport assay
- QUANTIFICATION AND STATISTICAL ANALYSIS

SUPPLEMENTAL INFORMATION

Supplemental Information includes five figures, one table, and two videos and can be found with this article online at https://doi.org/10.1016/j.celrep.2018.12.085.

ACKNOWLEDGMENTS

F.J.L.'s group is supported by a Wellcome Trust Senior Investigator Award (WT101052MA), the Alborada Trust's funding of the Alzheimer's Research UK Stem Cell Research Centre, and Dementias Platform UK. S.P.J. is a Wellcome Trust Senior Investigator. S.W. and J.H. received funding from the National Institute for Health Research University College London Hospitals Biomedical Research Centre and S.W. from an ARUK Senior Research Fellowship (ARUK-SRF2016B-2). D.L. is a Wellcome Trust Henry Dale fellow (86609). Research in S.P.J.'s and F.J.L.'s groups benefits from core support to the Gurdon Institute from the Wellcome Trust and Cancer Research UK.

AUTHOR CONTRIBUTIONS

F.P., L.D.E., D.L., and F.J.L. conceived and designed the experiments. F.P. and L.D.E. collected and analyzed the experimental data. F.P., L.D.E., R.S., and F.J.L. wrote the manuscript. J.H. and S.W. contributed cell lines. All authors edited and approved the final manuscript.

DECLARATION OF INTERESTS

The authors declare no competing interests.

Received: May 17, 2018
Revised: October 12, 2018
Accepted: December 18, 2018
Published: January 15, 2019

REFERENCES

Akhmanova, A., and Steinmetz, M.O. (2008). Tracking the ends: a dynamic protein network controls the fate of microtubule tips. Nat. Rev. Mol. Cell Biol. *9*, 309–322.

Alonso, Adel.C., Mederlyova, A., Novak, M., Grundke-Iqbal, I., and Iqbal, K. (2004). Promotion of hyperphosphorylation by frontotemporal dementia tau mutations. J. Biol. Chem. *279*, 34873–34881.

Applegate, K.T., Besson, S., Matov, A., Bagonis, M.H., Jaqaman, K., and Danuser, G. (2011). plusTipTracker: quantitative image analysis software for the measurement of microtubule dynamics. J. Struct. Biol. *176*, 168–184.

Ballatore, C., Lee, V.M.-Y., and Trojanowski, J.Q. (2007). Tau-mediated neurodegeneration in Alzheimer's disease and related disorders. Nat. Rev. Neurosci. *8*, 663–672.

Bence, N.F., Sampat, R.M., and Kopito, R.R. (2001). Impairment of the ubiquitin-proteasome system by protein aggregation. Science *292*, 1552–1555.

Bottanelli, F., Kromann, E.B., Allgeyer, E.S., Erdmann, R.S., Wood Baguley, S., Sirinakis, G., Schepartz, A., Baddeley, D., Toomre, D.K., Rothman, J.E., and Bewersdorf, J. (2016). Two-colour live-cell nanoscale imaging of intracellular targets. Nat. Commun. *7*, 10778.

Broers, J.L.V., Ramaekers, F.C.S., Bonne, G., Yaou, R.B., and Hutchison, C.J. (2006). Nuclear lamins: laminopathies and their role in premature ageing. Physiol. Rev. *86*, 967–1008.

Burke, B., and Stewart, C.L. (2013). The nuclear lamins: flexibility in function. Nat. Rev. Mol. Cell Biol. *14*, 13–24.

Caballero, B., Wang, Y., Diaz, A., Tasset, I., Juste, Y.R., Stiller, B., Mandelkow, E.-M., Mandelkow, E., and Cuervo, A.M. (2018). Interplay of pathogenic forms of human tau with different autophagic pathways. Aging Cell *17*, e12692.

Capell, B.C., and Collins, F.S. (2006). Human laminopathies: nuclei gone genetically awry. Nat. Rev. Genet. *7*, 940–952.

Chang, W., Worman, H.J., and Gundersen, G.G. (2015). Accessorizing and anchoring the LINC complex for multifunctionality. J. Cell Biol. *208*, 11–22.

Chou, C.-C., Zhang, Y., Umoh, M.E., Vaughan, S.W., Lorenzini, I., Liu, F., Sayegh, M., Donlin-Asp, P.G., Chen, Y.H., Duong, D.M., et al. (2018). TDP-43 pathology disrupts nuclear pore complexes and nucleocytoplasmic transport in ALS/FTD. Nat. Neurosci. *21*, 228–239.

Crisp, M., Liu, Q., Roux, K., Rattner, J.B., Shanahan, C., Burke, B., Stahl, P.D., and Hodzic, D. (2006). Coupling of the nucleus and cytoplasm: role of the LINC complex. J. Cell Biol. *172*, 41–53.

D'Souza, I., Poorkaj, P., Hong, M., Nochlin, D., Lee, V.M., Bird, T.D., and Schellenberg, G.D. (1999). Missense and silent tau gene mutations cause frontotemporal dementia with parkinsonism-chromosome 17 type, by affecting multiple alternative RNA splicing regulatory elements. Proc. Natl. Acad. Sci. U S A *96*, 5598–5603.

Dixit, R., Ross, J.L., Goldman, Y.E., and Holzbaur, E.L.F. (2008). Differential regulation of dynein and kinesin motor proteins by tau. Science *319*, 1086–1089.

Eftekharzadeh, B., Daigle, J.G., Kapinos, L.E., Coyne, A., Schiantarelli, J., Carlomagno, Y., Cook, C., Miller, S.J., Dujardin, S., Amaral, A.S., et al. (2018). Tau protein disrupts nucleocytoplasmic transport in Alzheimer's disease. Neuron *99*, 925–940.e7.

Fitzpatrick, A.W.P., Falcon, B., He, S., Murzin, A.G., Murshudov, G., Garringer, H.J., Crowther, R.A., Ghetti, B., Goedert, M., and Scheres, S.H.W. (2017). Cryo-EM structures of tau filaments from Alzheimer's disease. Nature *547*, 185–190.

Freibaum, B.D., Lu, Y., Lopez-Gonzalez, R., Kim, N.C., Almeida, S., Lee, K.-H., Badders, N., Valentine, M., Miller, B.L., Wong, P.C., et al. (2015). GGGGCC repeat expansion in C9orf72 compromises nucleocytoplasmic transport. Nature *525*, 129–133.

Frost, B. (2016). Alzheimer's disease: An acquired neurodegenerative laminopathy. Nucleus *7*, 275–283.

Frost, B., Bardai, F.H., and Feany, M.B. (2016). Lamin dysfunction mediates neurodegeneration in tauopathies. Curr. Biol. *26*, 129–136.

Fu, H., Hussaini, S.A., Wegmann, S., Profaci, C., Daniels, J.D., Herman, M., Emrani, S., Figueroa, H.Y., Hyman, B.T., Davies, P., and Duff, K.E. (2016). 3D visualization of the temporal and spatial spread of tau pathology reveals extensive sites of tau accumulation associated with neuronal loss and recognition memory deficit in aged tau transgenic mice. PLoS ONE *11*, e0159463.

Gerace, L., and Huber, M.D. (2012). Nuclear lamina at the crossroads of the cytoplasm and nucleus. J. Struct. Biol. *177*, 24–31.

Goedert, M., Ghetti, B., and Spillantini, M.G. (2012). Frontotemporal dementia: implications for understanding Alzheimer disease. Cold Spring Harb. Perspect. Med. *2*, a006254.

Götz, J., Probst, A., Spillantini, M.G., Schäfer, T., Jakes, R., Bürki, K., and Goedert, M. (1995). Somatodendritic localization and hyperphosphorylation of tau protein in transgenic mice expressing the longest human brain tau isoform. EMBO J. *14*, 1304–1313.

Grima, J.C., Daigle, J.G., Arbez, N., Cunningham, K.C., Zhang, K., Ochaba, J., Geater, C., Morozko, E., Stocksdale, J., Glatzer, J.C., et al. (2017). Mutant Huntingtin disrupts the nuclear pore complex. Neuron *94*, 93–107.e6.

Hernández-Vega, A., Braun, M., Scharrel, L., Jahnel, M., Wegmann, S., Hyman, B.T., Alberti, S., Diez, S., and Hyman, A.A. (2017). Local nucleation of microtubule bundles through tubulin concentration into a condensed tau phase. Cell Rep. *20*, 2304–2312.

Hoover, B.R., Reed, M.N., Su, J., Penrod, R.D., Kotilinek, L.A., Grant, M.K., Pitstick, R., Carlson, G.A., Lanier, L.M., Yuan, L.-L., et al. (2010). Tau mislocalization to dendritic spines mediates synaptic dysfunction independently of neurodegeneration. Neuron *68*, 1067–1081.

Hutton, M., Lendon, C.L., Rizzu, P., Baker, M., Froelich, S., Houlden, H., Pickering-Brown, S., Chakraverty, S., Isaacs, A., Grover, A., et al. (1998). Association of missense and 5′-splice-site mutations in tau with the inherited dementia FTDP-17. Nature *393*, 702–705.

Israel, M.A., Yuan, S.H., Bardy, C., Reyna, S.M., Mu, Y., Herrera, C., Hefferan, M.P., Van Gorp, S., Nazor, K.L., Boscolo, F.S., et al. (2012). Probing sporadic and familial Alzheimer's disease using induced pluripotent stem cells. Nature *482*, 216–220.

Jovičić, A., Mertens, J., Boeynaems, S., Bogaert, E., Chai, N., Yamada, S.B., Paul, J.W., 3rd, Sun, S., Herdy, J.R., Bieri, G., et al. (2015). Modifiers of C9orf72 dipeptide repeat toxicity connect nucleocytoplasmic transport defects to FTD/ALS. Nat. Neurosci. *18*, 1226–1229.

Kadavath, H., Hofele, R.V., Biernat, J., Kumar, S., Tepper, K., Urlaub, H., Mandelkow, E., and Zweckstetter, M. (2015). Tau stabilizes microtubules by binding at the interface between tubulin heterodimers. Proc. Natl. Acad. Sci. U S A *112*, 7501–7506.

Kelley, J.B., Datta, S., Snow, C.J., Chatterjee, M., Ni, L., Spencer, A., Yang, C.-S., Cubeñas-Potts, C., Matunis, M.J., and Paschal, B.M. (2011). The defective nuclear lamina in Hutchinson-Gilford progeria syndrome disrupts the nucleocytoplasmic Ran gradient and inhibits nuclear localization of Ubc9. Mol. Cell. Biol. *31*, 3378–3395.

Kowall, N.W., and Kosik, K.S. (1987). Axonal disruption and aberrant localization of tau protein characterize the neuropil pathology of Alzheimer's disease. Ann. Neurol. *22*, 639–643.

Larrieu, D., Britton, S., Demir, M., Rodriguez, R., and Jackson, S.P. (2014). Chemical inhibition of NAT10 corrects defects of laminopathic cells. Science *344*, 527–532.

Lee, V.M., Goedert, M., and Trojanowski, J.Q. (2001). Neurodegenerative tauopathies. Annu. Rev. Neurosci. *24*, 1121–1159.

Lewis, J., McGowan, E., Rockwood, J., Melrose, H., Nacharaju, P., Van Slegtenhorst, M., Gwinn-Hardy, K., Paul Murphy, M., Baker, M., Yu, X., et al. (2000). Neurofibrillary tangles, amyotrophy and progressive motor disturbance in mice expressing mutant (P301L) tau protein. Nat. Genet. *25*, 402–405.

Lim, J., and Yue, Z. (2015). Neuronal aggregates: formation, clearance, and spreading. Dev. Cell *32*, 491–501.

Luo, Y., Lee, I.-W., Jo, Y.-J., Namgoong, S., and Kim, N.-H. (2016). Depletion of the LINC complex disrupts cytoskeleton dynamics and meiotic resumption in mouse oocytes. Sci. Rep. *6*, 20408.

Mertens, J., Paquola, A.C.M., Ku, M., Hatch, E., Böhnke, L., Ladjevardi, S., McGrath, S., Campbell, B., Lee, H., Herdy, J.R., et al. (2015). Directly reprogrammed human neurons retain aging-associated transcriptomic signatures and reveal age-related nucleocytoplasmic defects. Cell Stem Cell *17*, 705–718.

Oberdoerffer, P., and Sinclair, D.A. (2007). The role of nuclear architecture in genomic instability and ageing. Nat. Rev. Mol. Cell Biol. *8*, 692–702.

Rossor, M.N., Fox, N.C., Mummery, C.J., Schott, J.M., and Warren, J.D. (2010). The diagnosis of young-onset dementia. Lancet Neurol. *9*, 793–806.

Schindelin, J., Arganda-Carreras, I., Frise, E., Kaynig, V., Longair, M., Pietzsch, T., Preibisch, S., Rueden, C., Saalfeld, S., Schmid, B., et al. (2012). Fiji: an open-source platform for biological-image analysis. Nat. Methods *9*, 676–682.

Shammas, S.L., Garcia, G.A., Kumar, S., Kjaergaard, M., Horrocks, M.H., Shivji, N., Mandelkow, E., Knowles, T.P.J., Mandelkow, E., and Klenerman, D. (2015). A mechanistic model of tau amyloid aggregation based on direct observation of oligomers. Nat. Commun. *6*, 7025.

Sheffield, L.G., Miskiewicz, H.B., Tannenbaum, L.B., and Mirra, S.S. (2006). Nuclear pore complex proteins in Alzheimer disease. J. Neuropathol. Exp. Neurol. *65*, 45–54.

Shi, Y., Kirwan, P., and Livesey, F.J. (2012a). Directed differentiation of human pluripotent stem cells to cerebral cortex neurons and neural networks. Nat. Protoc. *7*, 1836–1846.

Shi, Y., Kirwan, P., Smith, J., Robinson, H.P.C., and Livesey, F.J. (2012b). Human cerebral cortex development from pluripotent stem cells to functional excitatory synapses. Nat. Neurosci. *15*, 477–486.

Snow, C.J., Dar, A., Dutta, A., Kehlenbach, R.H., and Paschal, B.M. (2013). Defective nuclear import of Tpr in Progeria reflects the Ran sensitivity of large cargo transport. J. Cell Biol. *201*, 541–557.

Spillantini, M.G., and Goedert, M. (2013). Tau pathology and neurodegeneration. Lancet Neurol. *12*, 609–622.

Sposito, T., Preza, E., Mahoney, C.J., Setó-Salvia, N., Ryan, N.S., Morris, H.R., Arber, C., Devine, M.J., Houlden, H., Warner, T.T., et al. (2015). Developmental regulation of tau splicing is disrupted in stem cell-derived neurons from frontotemporal dementia patients with the 10 + 16 splice-site mutation in MAPT. Hum. Mol. Genet. *24*, 5260–5269.

Thies, E., and Mandelkow, E.-M. (2007). Missorting of tau in neurons causes degeneration of synapses that can be rescued by the kinase MARK2/Par-1. J. Neurosci. *27*, 2896–2907.

Trinczek, B., Ebneth, A., Mandelkow, E.M., and Mandelkow, E. (1999). Tau regulates the attachment/detachment but not the speed of motors in microtubule-dependent transport of single vesicles and organelles. J. Cell Sci. *112*, 2355–2367.

von Bergen, M., Barghorn, S., Biernat, J., Mandelkow, E.-M., and Mandelkow, E. (2005). Tau aggregation is driven by a transition from random coil to beta sheet structure. Biochim. Biophys. Acta *1739*, 158–166.

Wang, Y., and Mandelkow, E. (2016). Tau in physiology and pathology. Nat. Rev. Neurosci. *17*, 5–21.

Wang, J.-Z., Xia, Y.-Y., Grundke-Iqbal, I., and Iqbal, K. (2013). Abnormal hyperphosphorylation of tau: sites, regulation, and molecular mechanism of neurofibrillary degeneration. J. Alzheimers Dis. *33* (*Suppl 1*), S123–S139.

Webster, M., Witkin, K.L., and Cohen-Fix, O. (2009). Sizing up the nucleus: nuclear shape, size and nuclear-envelope assembly. J. Cell Sci. *122*, 1477–1486.

Witman, G.B., Cleveland, D.W., Weingarten, M.D., and Kirschner, M.W. (1976). Tubulin requires tau for growth onto microtubule initiating sites. Proc. Natl. Acad. Sci. U S A *73*, 4070–4074.

Zempel, H., and Mandelkow, E.-M. (2015). Tau missorting and spastin-induced microtubule disruption in neurodegeneration: Alzheimer disease and hereditary spastic paraplegia. Mol. Neurodegener. *10*, 68.

Zhang, K., Donnelly, C.J., Haeusler, A.R., Grima, J.C., Machamer, J.B., Steinwald, P., Daley, E.L., Miller, S.J., Cunningham, K.M., Vidensky, S., et al. (2015). The C9orf72 repeat expansion disrupts nucleocytoplasmic transport. Nature *525*, 56–61.

Zhang, Y.-J., Gendron, T.F., Grima, J.C., Sasaguri, H., Jansen-West, K., Xu, Y.-F., Katzman, R.B., Gass, J., Murray, M.E., Shinohara, M., et al. (2016). C9ORF72 poly(GA) aggregates sequester and impair HR23 and nucleocytoplasmic transport proteins. Nat. Neurosci. *19*, 668–677.

Zhang, K., Daigle, J.G., Cunningham, K.M., Coyne, A.N., Ruan, K., Grima, J.C., Bowen, K.E., Wadhwa, H., Yang, P., Rigo, F., et al. (2018). Stress granule assembly disrupts nucleocytoplasmic transport. Cell *173*, 958–971.e17.

OPEN
ACCESS
CellPress

STAR★METHODS

KEY RESOURCES TABLE

REAGENT or RESOURCE	SOURCE	IDENTIFIER
Antibodies		
4R-tau; rabbit	Cosmo Bio	Cat#CAC-TIP-4RT-P01; RRID:N/A
Alexa Fluor 488 donkey anti-rabbit	Thermo Fisher Scientific	Cat#A21206; RRID:AB_2535792
Alexa Fluor 488 goat anti-chicken	Thermo Fisher Scientific	Cat#A11039; RRID:AB_142924
Alexa Fluor 594 donkey anti-mouse	Thermo Fisher Scientific	Cat#A21203; RRID:AB_141633
Alexa Fluor 647 donkey anti-rabbit	Thermo Fisher Scientific	Cat#A31573; RRID:AB_2536183
Alexa Fluor 647 goat anti-rat	Thermo Fisher Scientific	Cat#A21247; RRID:AB_141778
CTIP2; rat monoclonal	Abcam	Cat#ab18465; RRID:AB_10015215
GFP; chicken polyclonal	Abcam	Cat#ab13970; RRID:AB_300798
IRDye 680 RD donkey anti-mouse	LI-COR bioscience	Cat#925-68072; RRID:N/A
IRDye 800 CW donkey anti-rabbit	LI-COR bioscience	Cat#926-32213; RRID:AB_621848
LaminB1 [119D5-F1]; mouse monoclonal	Abcam	Cat#ab8982; RRID:AB_1640627
LaminB1; rabbit polyclonal	Abcam	Cat#ab16048; RRID:AB_443298
MAP2; chicken polyclonal	Abcam	Cat#ab5392; RRID:AB_2138153
NUP98; rat monoclonal	Abcam	Cat#ab50610; RRID:AB_881769
Phospho-tau pS202/T205 - AT8; mouse monoclonal	Thermo Fisher Scientific	Cat#MN1020; RRID:AB_223647
Phospho-tau S404; rabbit monoclonal	Abcam	Cat#ab92676; RRID:AB_10561457
tau RD3; mouse monoclonal	Millipore	Cat#05-803; RRID:AB_310013
Tbr1; rabbit polyclonal	Abcam	Cat#ab31940; RRID:AB_2200219
total tau HT7; mouse monoclonal	Thermo Fisher Scientific	Cat#MN1000; RRID:AB_2314654
total tau; rabbit polyclonal	Dako Cytomation	Cat#A0024; RRID:AB_10013724
β-actin; mouse monoclonal	Sigma	Cat#A2228; RRID:AB_476697
β3-tubulin; mouse monoclonal	BioLegends	Cat#MMS-435P; RRID:AB_2313773
Biological Samples		
Human brain FFPE sections from frontal and temporal cortex (cohort 1)	Queen's Square Brain Bank, Institute of Neurology, University College London	Tissue request MTA ID 20170112_UCL
Human brain FFPE sections from frontal and temporal cortex (cohort 2)	London Neurodegenerative Diseases Brain Bank and Brains for Dementia Research	Tissue request No 1827
Chemicals, Peptides, and Recombinant Proteins		
2-Mercaptoethanol	Thermo Fisher Scientific	Cat#21985-023
Accutase	Innovative Cell Technologies	Cat#AT104
B-27 supplement	Thermo Fisher Scientific	Cat#17504-044
Bovine Serum Albumin (BSA)	Sigma	Cat#A2153
cOmplete, Mini, EDTA-free Protease Inhibitor Cocktail Tablets	Sigma	Cat#04693159001
Colloidal Blue Staining Kit	Thermo Fisher Scientific	Cat#LC6025
DAB Peroxidase (HRP) Substrate Kit	Vector Laboratories	Cat#SK-4100
DAPI	Sigma	Cat#D9542
Dispase	Thermo Fisher Scientific	Cat#17105
DL-Dithiothreitol solution	Sigma	Cat#646563
DMEM/F-12, GlutaMAX	Thermo Fisher Scientific	Cat#31331-028
DNase I	New England BioLabs	Cat#M0303S

(*Continued on next page*)

Continued

REAGENT or RESOURCE	SOURCE	IDENTIFIER
Dimethyl sulfoxide (DMSO)	Sigma	Cat#D2650
Donkey serum	Abcam	Cat#ab7475
Dorsomorphin dihydrochloride	Tocris	Cat#3093
Essential 8 medium	Thermo Fisher Scientific	Cat#A1517001
Fibroblast growth factor 2 (FGF2)	PeproTech	Cat#100-18B
Halt Phosphatase Inhibitor Cocktail	Thermo Fisher Scientific	Cat#78420
High Capacity cDNA Reverse Transcription kit	Thermo Fisher Scientific	Cat#4368814
Insulin	Sigma	Cat#19278
L-Glutamine	Thermo Fisher Scientific	Cat#25030-024
Laminin	Sigma	Cat#L2020
N-2 supplement	Thermo Fisher Scientific	Cat#17502-048
N-Lauroylsarcosine sodium salt solution	Sigma	Cat#61747
Neurobasal	Thermo Fisher Scientific	Cat#12348-017
Nocodazole	Tocris	Cat#1228
Non-essential amino acid solution	Thermo Fisher Scientific	Cat#11140-050
NuPAGE LDS Sample Buffer	Thermo Fisher Scientific	Cat#NP0007
Paraformaldehyde (PFA)	Sigma	Cat#158127
PBS	N/A	N/A
Penicillin-streptomycin	Thermo Fisher Scientific	Cat#15140-122
Phenylmethanesulfonyl fluoride (PMSF) solution	Sigma	Cat#93482
ProLong Gold Antifade Mountant	Thermo Fisher Scientific	Cat#P36930
RIPA Buffer	Sigma	Cat#R0278
SB431542	Tocris Bioscience	Cat#1614
Sodium pyruvate	Sigma	Cat#S8636
Sudan Black B	Sigma	Cat# 199664
SYBR Green JumpStart Taq Ready Mix	Sigma	Cat#S4438
Triton X-100	Sigma	Cat#T8787
TRIzol	Thermo Fisher Scientific	Cat#15596026
Tween 20	Sigma	Cat#P9416
Trisodium citrate dihydrate	Sigma	Cat#S1804
Critical Commercial Assays		
Precision Red Advanced Protein Assay	Cytoskeleton, Inc.	Cat# ADV02-A
Experimental Models: Cell Lines		
Human: non-demented control (NDC) iPSC line	Israel et al., 2012	N/A
Human: MAPT IVS10+16-A and MAPT IVS10+16-B iPSC lines	Sposito et al., 2015	N/A
Human: MAPT P301L and MAPT P301L-isogenic iPSC lines	Janssen Pharmaceutica	IMI STEMBANCC project agreement ICD 483960
Oligonucleotides		
4RMAPT For 5′ – AAGATCGGCTCCACTGAGAA – 3′	This paper	N/A
4RMAPT Rev 5′ – CACACTTGGACTGGACGTTG – 3′	This paper	N/A
GAPDH For 5′ – AATGAAGGGGTCATTGATGG – 3′	This paper	N/A
GAPDH Rev 5′ – AAGGTGAAGGTCGGAGTCAA – 3′	This paper	N/A
RPS9 For 5′ – CAGCTTCATCTTGCCCTCAT – 3′	This paper	N/A
RPS9 Rev 5′ – CTGCTGACGCTTGATGAGAA – 3′	This paper	N/A

(*Continued on next page*)

Continued		
REAGENT or RESOURCE	SOURCE	IDENTIFIER
Recombinant DNA		
dsEGFP-EB3-7	Michael Davidson	Addgene plasmid # 56474; RRID: Addgene_56474
pLVX-EF1alpha-2xGFP:NES-IRES-2xRFP:NLS	Mertens et al., 2015	Addgene plasmid #71396; RRID: Addgene_71396
Software and Algorithms		
ABI StepOnePlus software	Thermo Fisher Scientific	N/A; RRID:N/A
Fiji	Schindelin et al., 2012	RRID:SCR_002285
Image Studio Lite	LI-COR	**RRID**:SCR_013715
Mascot	MATRIX Science	RRID:SCR_014322
plusTipTracker	Applegate et al., 2011	N/A; RRID:N/A
Prism 6	GraphPad	RRID:SCR_002798

CONTACT FOR REAGENT AND RESOURCE SHARING

Further information and requests for resources and reagents should be directed to and will be fulfilled by the Lead Contact, Rick Livesey (r.livesey@ucl.ac.uk).

EXPERIMENTAL MODEL AND SUBJECT DETAILS

Human iPSC lines

MAPT IVS10+16-A and *MAPT* IVS10+16-B mutant iPSCs were as reported in (Sposito et al., 2015). *MAPT* P301L was generated from Janssen Pharmaceutica by TALEN editing the line *MAPT* P301-isogenic, under the IMI STEMBANCC project agreement ICD 483960. The non-demented control line was previously reported (Israel et al., 2012). iPSC cells were growth and expanded in feeder-free conditions using Essential 8 Medium (Thermo Fisher Scientific), at 37°C with 5% CO_2. Essential 8 Medium was replaced daily.

Human post-mortem brain sections

Human brain sections were obtained from the Queen's Square Brain Bank, Institute of Neurology, University College London (cohort one) and from the London Neurodegenerative Diseases Brain Bank and Brains for Dementia Research (cohort two). For cohort one, control brains included one male (age 71) and one female (age 56). FTD-MAPT IVS10+16 brains were from two males (age 52 and 66). For cohort two, control brains were from five males (age 63-77) and one female (age 43). FTD-MAPT IVS10+16 brains were from four males (age 48-71) and two females (age 58 and 63). The use of human post-mortem tissues for this study has been approved with Research Ethics Committee reference ID 08/H0718/54+5 for cohort one and reference ID 08/MRE09/38+5 for cohort two.

METHOD DETAILS

Generation of iPSC-derived cortical neurons and drug treatments

Differentiation of iPSCs to cortical neurons was carried out as described, with minor modifications (Shi et al., 2012b; 2012a). Briefly, dissociated iPSCs were plated on Geltrex (Thermo Fisher Scientific)-coated plates to reach full confluence. Neural induction was initiated the next day (Day 0) by changing the culture medium to a 1:1 mixture of DMEM/N-2 (DMEM/F-12 GlutaMAX; 1 × N-2; 5 μg ml^{-1} insulin; 1 mM L-glutamine; 100 μm non-essential amino acids; 100 μM β-mercaptoethanol; 50 U ml^{-1} penicillin and 50 mg ml^{-1} streptomycin) and Neurobasal/B-27 (Neurobasal; 1 × B-27; 200 mM L-glutamine; 50 U ml^{-1} penicillin and 50 mg ml^{-1} streptomycin) media (hereafter referred as N2B27) supplemented with 1 μM dorsomorphin and 10 μM SB431542 to inhibit TGFβ signaling and support neuronal differentiation and neurogenesis, media was replaced every 24 hours. At day 12 neuroepithelial sheet was harvested and dissociated using the enzyme Dispase and replated on laminin-coated plates. The day after, media was replaced with N2B27 containing 20 ng/mL FGF2. N2B27+FGF2 was added freshly daily for 4 days to promote the maturation of neural rosettes. After 4 days FGF2 was withdrawn and neural rosettes were maintained in N2B27 refreshing medium every other day. At day 30 neural rosettes were dissociated using Accutase and neural progenitor cells were plated on laminin-coated plates at 150,000 cells/mm^2. Plated neurons were maintained for up to 120 days with a medium change every other day. To establish identity and quality of cortical neuronal inductions, gene expression profiling was performed on a custom gene expression panel. RNA was isolated from induced cortical neurons using TRIzol (Thermo Fisher Scientific), according to the manufacturer's instructions. Expression levels of mRNAs enriched in deep and upper layer cortical neurons were assessed using a nanoString (nanoString Technologies) gene expression panel of approximately 250 genes. After subtracting the maximum negative control

probe counts, gene counts were normalized using the geometric mean of 6 positive control probes and of 7 housekeeping genes (CLTC, GAPDH, GUSB, PPIA, RPLP1, RPS15A, RPS9). For nocodazole (Tocris) treatment, neurons were grown for 120 days *in vitro* (DIV) and compound was added at 33 μM for 2 hours before imaging. DMSO was used as vehicle.

Protein extraction and western blot analysis

Total cell protein was extracted using RIPA buffer (Sigma) supplemented with protease inhibitors (Sigma) and Halt phosphatase inhibitors (Thermo Fisher Scientific). Protein quantification was performed using Precision Red Advanced Protein Assay buffer (Cytoskeleton, Inc.). For each sample, 30 μg of protein were mixed with 1X NuPAGE LDS Sample Buffer (Thermo Fisher Scientific) + 1 μM Dithiothreitol. Samples were heated at 100°C for 10 minutes and loaded on NuPAGE 4%–12% Bis-Tris gel (Thermo Fisher Scientific). Afterward, proteins were transferred on PVDF membrane (Millipore) for 1 h at 100 V. Membranes were blocked for another 60 min in 5% BSA in PBST (PBS containing 0.05% Tween 20). All primary antibodies were incubated overnight in 5% milk in PBST at 4°C. Next day, membranes were incubated for at least 1 h in secondary antibody and washed gently in PBST buffer for further 30-60 min. Immunoblots were detected using LI-COR Odyssey CLx Infrared Imaging System and processed with the Image Studio Software (LI-COR).

Sarkosyl extraction

iPSC-derived neurons (120 DIV) were homogenized in Tris-NaCl buffer (25 mM TrisHCl, 150 mM NaCl, 1 mM EDTA, 1 mM EGTA, 5 mM $Na_4P_2O_7$, pH 7.6) supplemented with 1 mM PMSF, protease inhibitors (Sigma) and Halt phosphatase inhibitors (Thermo Fisher Scientific). Homogenate was subjected to ultracentrifugation at 150,000 g for 30 min at 4°C. The pellet was re-suspended in an equal volume of 10 mM TrisHCl, 0.8 M NaCl, 10% sucrose, pH 7.6 supplemented with 1 mM PMSF, protease inhibitors and Halt phosphatase inhibitors. The re-suspended pellet was centrifuged at 20,000 g for 30 min at 4°C, the supernatant was incubated with 1% sarkosyl (N-lauroylsarkosine sodium salt; Sigma) for 1 hour at room temperature and ultracentrifuged at 150,000 g for 30 min at 4°C. The resulting pellet (sarkosyl insoluble fraction) was resuspended in 1X NuPAGE LDS Sample Buffer (Thermo Fisher Scientific) + 1 mM Dithiothreitol. Samples were heated at 100°C for 10 minutes and loaded on NuPAGE 4%–12% Bis-Tris gel (Thermo Fisher Scientific).

Immunoprecipitation and mass spectrometric analysis of intracellular tau

Tau was immunoprecipitated from 1 mg of total protein extracted from iPSC-derived neurons (120 DIV) using a polyclonal anti-tau antibody (Dako Cytomation). Immunoprecipitated samples were analyzed by western blot using a monoclonal tau antibody (MN1000; Thermo Fisher Scientific) or stained with colloidal blue (Thermo Fisher Scientific). Bands that corresponded to tau by western blot analysis were excised from the colloidal blue SDS-PAGE. Peptide masses of digested protein samples were determined using a Bruker ultrafleXtreme Maldi mass spectrometer in reflectron mode and ms/ms fragmentation performed in LIFT mode. Data analysis was with FlexAnalysis, BioTools and ProteinScape software (Bruker). Database searches of the combined mass fingerprint-ms/ms data were performed using Mascot (http://www.matrixscience.com).

RNA extraction and qRT-PCR analysis

Total RNA was extracted using TRIzol according to manufacturer protocol (Invitrogen). 1 μg of RNA was treated with DNase I (New England BioLabs) and 500 ng were retrotranscribed using the High Capacity cDNA Reverse Transcription kit (Applied Biosystems). qRT-PCR were performed in a StepOnePlus instrument (Applied Biosystems) using the SYBR Green JumpStart Taq Ready Mix (Sigma) in a final volume of 15 μl, using the following protocol: 95°C for 2 min, 40 cycles at 94°C for 15 s, 60°C for 30 s. *MAPT* mRNA expression was assessed relative to *GAPDH* and *RPS9* housekeeping genes using the specific primers reported in the Key Resource Table. Results were analyzed using the ABI StepOnePlus software (Thermo Fisher Scientific).

Confocal microscopy and image analysis

For immunofluorescent labeling and imaging, cells were washed 3 times in PBS and then fixed using 4% paraformaldehyde (v/v) in PBS for 15 minutes at RT. After 3 washes in PBS, cells were permeabilised in PBS+0.3% Triton X-100 (Sigma; Tx) for 15 minutes at room temperature (RT). After 3 washes in PBS, cells were blocked using 5% donkey serum in PBS+0.3% Triton X-100 (PBS-Tx+5% DS) for 1 hr at RT. For AT8-tau immunostaining (ThermoFisher; MN1020), 5% BSA (Sigma) (w/v) in PBS+0.3% Triton X-100 (PBS-Tx+5% BSA) was used as a blocking agent. Primary antibodies were diluted in PBS-Tx+5% DS or PBS-Tx+5% BSA as indicated below and incubated overnight at 4°C. Cells were washed 3 times in PBS and incubated 1 hr in the dark at RT with secondary antibodies diluted 1:1000 in PBS-Tx+5% DS or PBS-Tx+5% BSA. After 3 washes in PBS, samples were incubated for 5 minutes at RT with DAPI diluted 1:5000 in PBS and then washed 3 additional times with PBS. Samples were mounted using ProLong Gold antifade (Thermo Fisher Scientific).

Standard confocal images were acquired with an Olympus Inverted FV1000 confocal microscope and processed using Fiji software (Schindelin et al., 2012). STED imaging was performed on a custom built, dual color, beam scanning system with gated detection optically identical to the instrument described in (Bottanelli et al., 2016).

For image analysis of colocalization of tau and MAP2, Pearson's R correlation was calculated using the Coloc2 plugin for Fiji (https://imagej.net/Coloc2). To quantify nuclear invaginations neurons were co-stained for LaminB1 and DAPI. Nuclear lamina signal was assigned as either nuclear boundary or invaginated (i.e., within the DAPI stained area defining the nucleus) using a custom plugin

for the Fiji bioimage analysis software. Nuclei with a proportion of invaginated laminB1 that exceeded 0.3 were considered as invagination positive (see Figure 3B and S3 for details). At least 5 imaging fields from three independent experiments for genotype were analyzed.

Staining of human formalin-fixed paraffin embedded (FFPE) brain sections

FFPE slides were deparaffinised and rehydrated, then boiled for 20 minutes in 10 mM Tri-sodium citrate buffer, pH6 + 0.05% Tween 20, for antigen retrieval. Slides were cooled to RT before staining. 3,3′-Diaminobenzidine (DAB) staining was performed using the DAB Peroxidase (HRP) Substrate Kit (Vector Laboratories) according to manufacturer's instructions. After DAB staining, whole sections were imaged using an Axio Scan.Z1 microscope (Zeiss). For immunofluorescence, after antigen retrieval slides were blocked using 5% BSA in PBS+0.3% Triton X-100 (PBS-Tx+5% BSA) for 20 minutes at RT. Primary antibodies were diluted in PBS-Tx+5% BSA as indicated below and incubated O/N at 4°C in humidified chamber. Slides were washed 3 times in PBS and incubated for 30 minutes in the dark at RT with secondary antibodies diluted 1:1000 in PBS-Tx+5% BSA. After 3 washes in PBS, samples were incubated 5 minutes with DAPI diluted 1:5000 in PBS. After 3 washes in PBS, slides were incubated for 20 minutes with 0.01% Sudan Black B (Sigma) in 70% ethanol. Samples were mounted using ProLong Gold antifade (Thermo Fisher Scientific). Images were acquired through Olympus Inverted FV1000 confocal microscope and processed using the Fiji software.

For cohort one, nuclear lamina invaginations were quantified after 3,3′-Diaminobenzidine (DAB) staining of LaminB1. Nuclei were scored from 20 randomly acquired imaging fields from each individual. Nuclei were considered positive (folded) if invaginations extended into the nuclear interior for at least 3 μm. The percentage of folded respect to total nuclei was calculated for each imaging field. For cohort two, neurons were co-stained for LaminB1 and DAPI. Nuclear lamina signal was assigned as either nuclear boundary or invaginated (i.e., within the DAPI stained area defining the nucleus) using custom plugin for the Fiji bioimage analysis software. Nuclei with a proportion of invaginated laminB1 that exceeded 0.3 were considered as invagination positive (see Figure 3B and S3 for details).

Live imaging of microtubule dynamics

Neurons were grown to 100 DIV in individual μ-Dish 35 mm dishes (Ibidi) and transfected with a plasmid encoding for GFP-EB3 (gift from Michael Davidson; Addgene plasmid # 56474). 48h after transfection, neurons were subjected to live imaging using a Leica SP5 microscope equipped with a controlled environment chamber (37°C; 5% CO_2). Images were acquired at resonant scanning with a 63x objective (1frame/sec). Resulting movies were analyzed using the plusTipTracker software (Applegate et al., 2011).

Nucleocytoplasmic transport assay

Nucleocytoplasmic trafficking was analyzed by infection of 120 DIV human iPSC-derived neurons with the pLVX-EF1alpha-2xGFP:NES-IRES-2xRFP:NLS construct (Addgene plasmid #71396; Mertens et al., 2015). After 6 days, neurons were fixed and immunostained for β3-tubulin and GFP. Only cells positive for neuron-specific β3-tubulin were considered. The nuclear to cytoplasmic ratios of both GFP and RFP (nucRFP:cytRFP and nucGFP:cytGFP) were calculated separately using the integrated density of ROIs drawn within and outside the nucleus (see Fig.S5 for details).

QUANTIFICATION AND STATISTICAL ANALYSIS

Unless otherwise specified, data are presented as mean values of the number of independently conducted experiments indicated in the legend of each figure. Error bars represent the standard error of mean (SEM). Statistical analysis was performed using the Prism6 analytical software (GraphPad). Unpaired Student's t test was used to compare differences between two groups, assuming the data were normally distributed. One-way ANOVA followed by Tukey's or Dunnett's correction for multiple testing (as indicated in figure legends) was used to analyze the differences between more than two groups. *** $p < 0.01$, **$p < 0.01$, *$p < 0.05$.

Cell Reports, Volume *26*

Supplemental Information

Microtubules Deform the Nuclear Membrane and Disrupt Nucleocytoplasmic Transport in Tau-Mediated Frontotemporal Dementia

Francesco Paonessa, Lewis D. Evans, Ravi Solanki, Delphine Larrieu, Selina Wray, John Hardy, Stephen P. Jackson, and Frederick J. Livesey

Figure S1

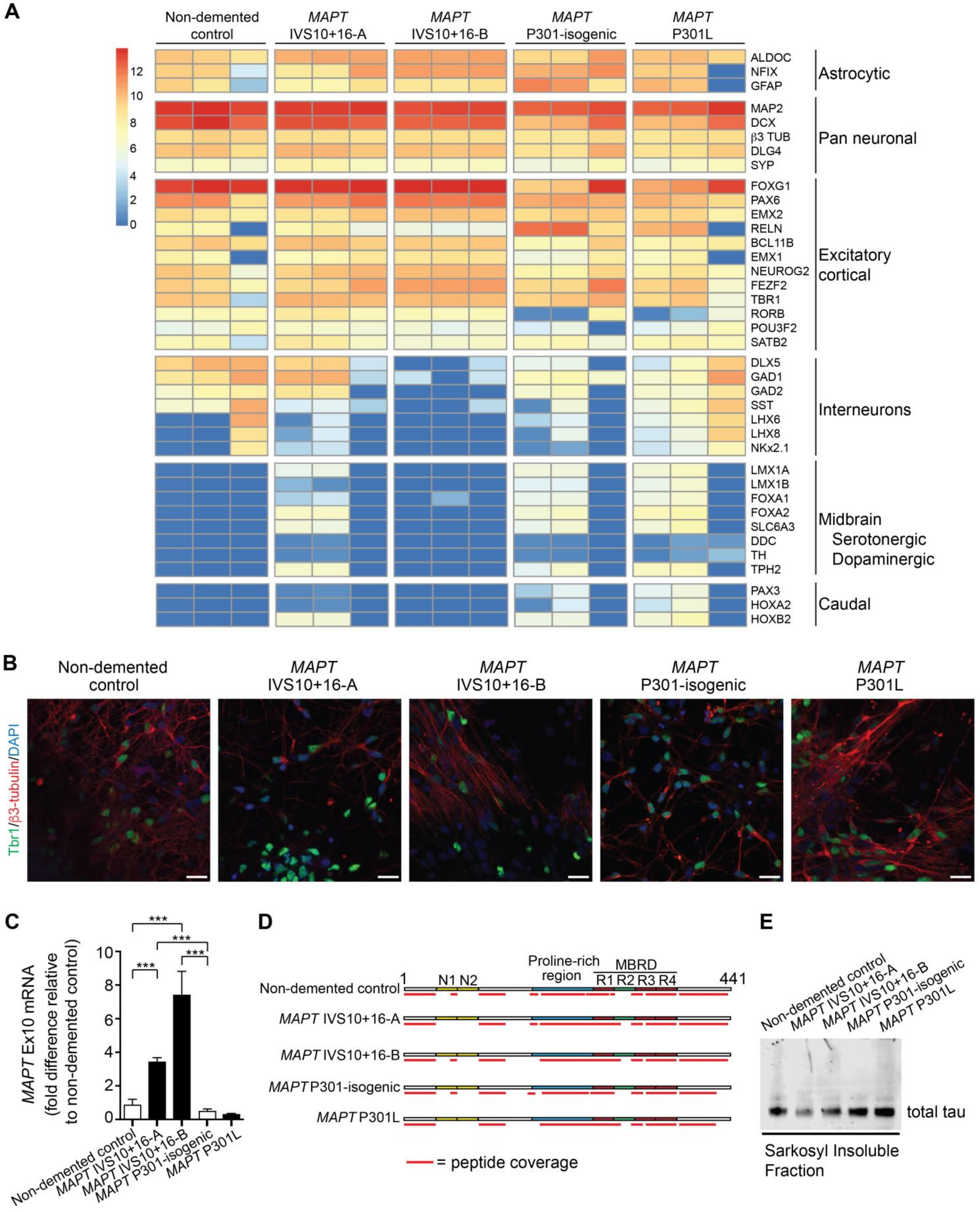
A
Non-demented control
MAPT IVS10+16-A
MAPT IVS10+16-B
MAPT P301-isogenic
MAPT P301L
0
2
4
6
8
10
12
ALDOC
NFIX
GFAP
Astrocytic
MAP2
DCX
β3 TUB
DLG4
SYP
Pan neuronal
FOXG1
PAX6
EMX2
RELN
BCL11B
EMX1
NEUROG2
FEZF2
TBR1
RORB
POU3F2
SATB2
Excitatory cortical
DLX5
GAD1
GAD2
SST
LHX6
LHX8
NKx2.1
Interneurons
LMX1A
LMX1B
FOXA1
FOXA2
SLC6A3
DDC
TH
TPH2
Midbrain
Serotonergic
Dopaminergic
PAX3
HOXA2
HOXB2
Caudal
B
Non-demented control
MAPT IVS10+16-A
MAPT IVS10+16-B
MAPT P301-isogenic
MAPT P301L
Tbr1/β3-tubulin/DAPI
C
MAPT Ex10 mRNA (fold difference relative to non-demented control)
0
2
4
6
8
10
***
***
***
***
Non-demented control
MAPT IVS10+16-A
MAPT IVS10+16-B
MAPT P301-isogenic
MAPT P301L
D
1
441
N1 N2
Proline-rich region
MBRD
R1 R2 R3 R4
Non-demented control
MAPT IVS10+16-A
MAPT IVS10+16-B
MAPT P301-isogenic
MAPT P301L
= peptide coverage
E
Non-demented control
MAPT IVS10+16-A
MAPT IVS10+16-B
MAPT P301-isogenic
MAPT P301L
total tau
Sarkosyl Insoluble Fraction

Related to Figure 1; Figure S1. Generation and characterization of human iPSC-derived neurons from familial FTD-*MAPT*.

(A) Heat map showing the relative expression of 38 genes (clustered by cell type) from FTD-MAPT neurons (*MAPT* IVS10+16-A/B and *MAPT* P301L) and non-demented and MAPT P301 isogenic control neurons (iPSC-derived neurons at 120 DIV; three biological replicates). **(B)** Immunofluorescences of iPSC-derived cerebral cortex excitatory neurons generated from control (non-demented control and *MAPT* P301-isogenic) and familial FTD-*MAPT* (*MAPT* IVS10+16-A/B and *MAPT* P301L), expressing the cortical layer 6 transcription factor Tbr1 (green) and β3-tubulin (red). DNA counterstain (DAPI, blue) is also shown. Scale bar = 20 μm. **(C)** qRT-PCR showing the relative abundance of *MAPT* mRNA containing the exon 10 (encoding for the additional microtubule binding repeat). FDT-*MAPT* (red) neurons are shown with respect to their respective controls (green). GAPDH and RPS9 were used as housekeeping genes (one-way ANOVA followed by Tukey's test, *** = P<0.001; error bar represents s.e.m.; n = 3 independent experiments). **(D)** Schematic of mass spectrometry peptide coverage (red line) for tau protein following immunoprecipitation of neuronal cell lysates from FTD-*MAPT* and control neurons (same genotypes as A; 120 DIV). 1 and 441 represent the NH- and COOH- protein extremities respectively. The position of alternatively spliced N1 and N2 domains (yellow), the proline rich domain (blue). The microtubule binding region domain (MBRD) has four microtubule binding regions R1,3 and 4 are shown in green, while the alternatively spliced R2 is shown in red. **(E)** Total tau detected in the sarkosyl-insoluble fraction across the indicated genotypes. No significant difference was detected in the amount of tau detected from neurons of each genotype.

Figure S2

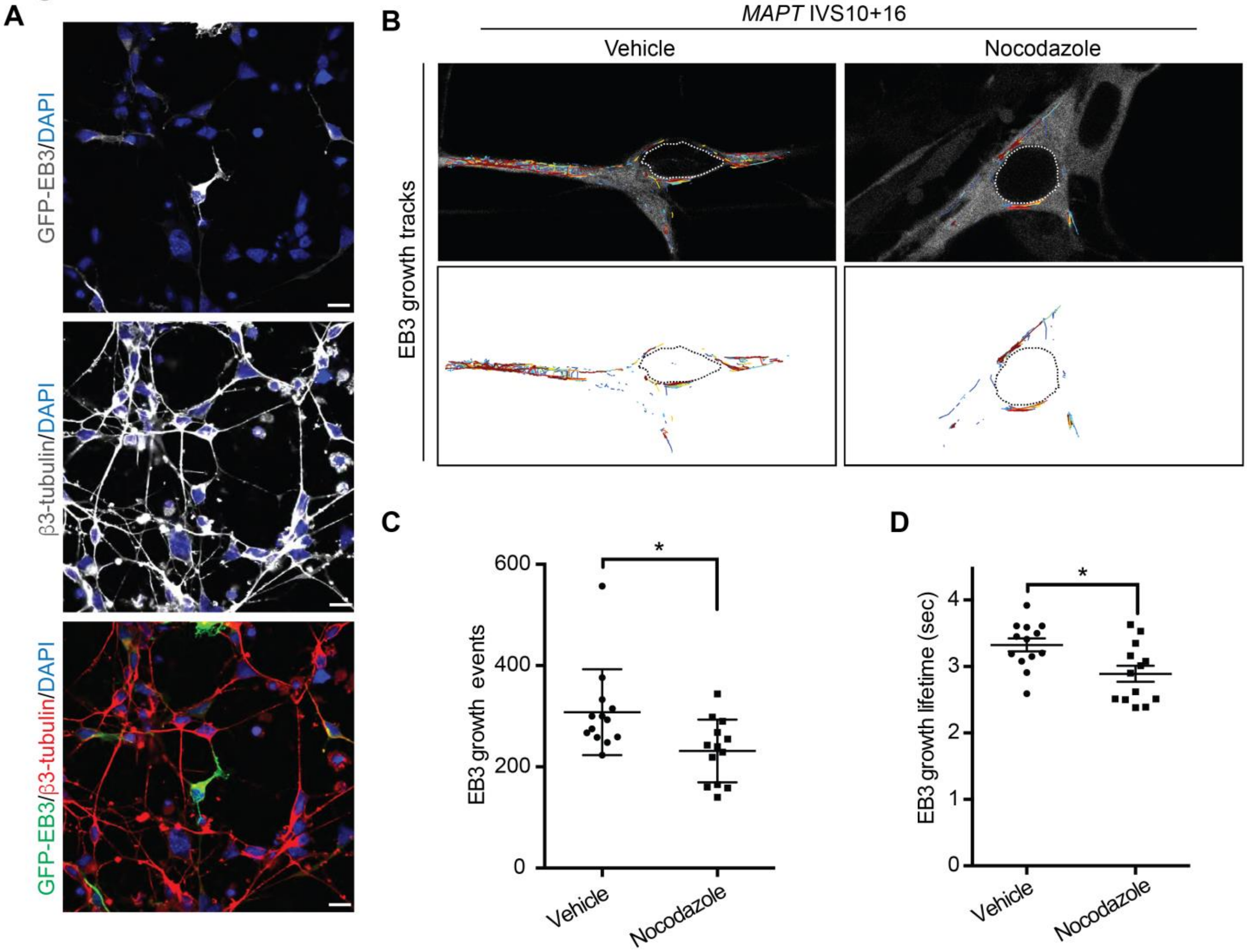

A
GFP-EB3/DAPI
β3-tubulin/DAPI
GFP-EB3/β3-tubulin/DAPI
B
MAPT IVS10+16
Vehicle
Nocodazole
EB3 growth tracks
C
EB3 growth events
600
400
200
0
*
Vehicle
Nocodazole
D
EB3 growth lifetime (sec)
4
3
2
1
0
*
Vehicle
Nocodazole

Related to Figure 2; Figure S2. Expression of GFP-EB3 recombinant protein in human iPSC-derived neurons.

(A) Immunofluorescence of iPSC-derived neurons (non-demented control; 120DIV) expressing GFP-EB3 protein, following (48 hr) lentivirus transfection. Left panel shows β3-tubulin (grey) and DAPI (blue). Middle panel shows β3-tubulin (grey) and DAPI (blue). Right panel shows merge of GFP-EB3 (green), β3-tubulin (red) and DAPI (blue). Scale bar = 10 µm. **(B)** Total microtubule trajectories (200-second interval; identified using plusTip track software) demonstrate reduction of EB3 comets after nocodazole treatment in FTD-MAPT neurons (MAPT IVS10+16-A; 120 DIV), overlaid on stills from GFP-EB3 live imaging (grey), white dotted line indicates the nucleus. **(C)** Quantification of reduction in EB3 growth events and **(D)** EB3 growth life time after two hours of nocodazole treatment. Pair-wise comparison of the vehicle and nocodazole treated neurons was performed using Student's *t* test; *=$P<0.05$; error bar represents s.e.m.; n >10 cells).

Figure S3

A

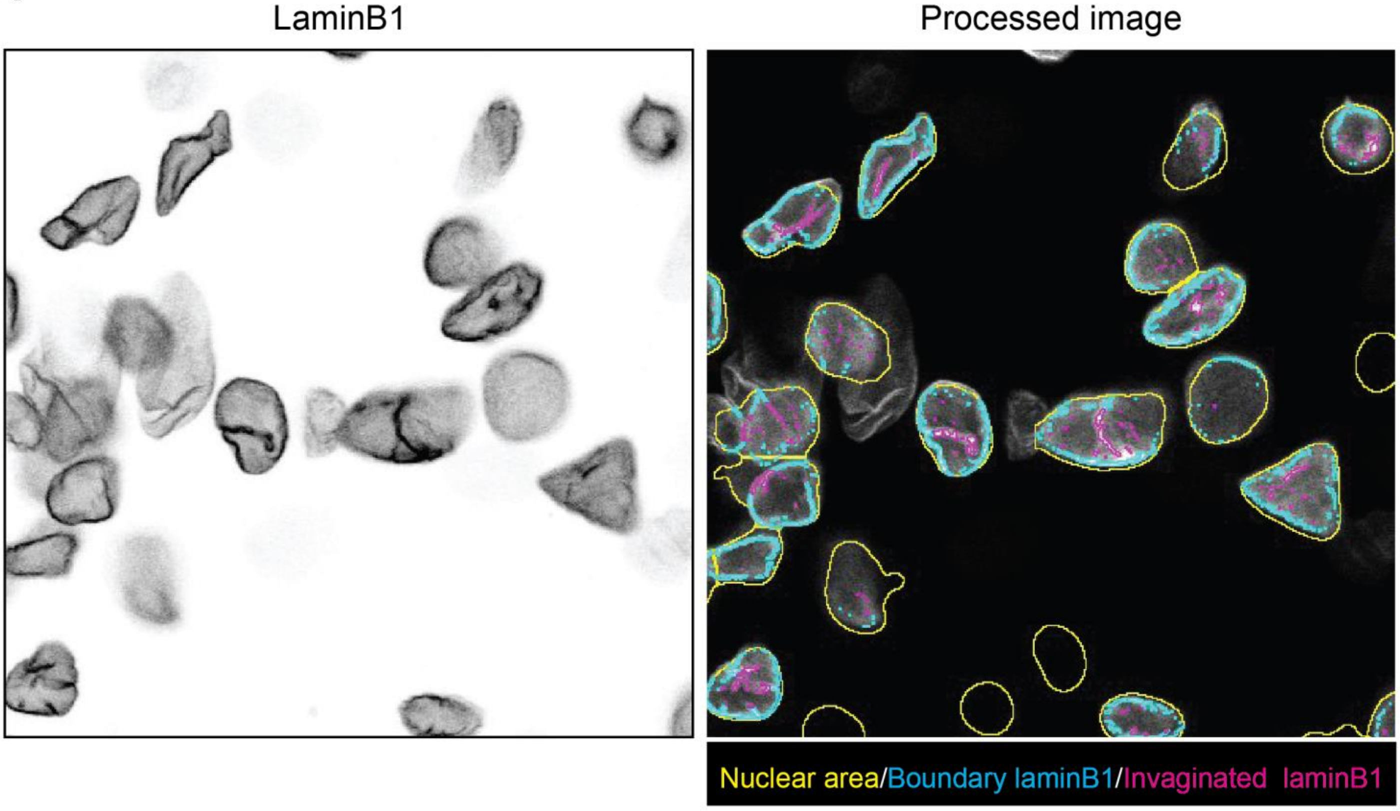

Boundary laminB1 + Invaginated laminB1 = Total laminB1

Invaginated laminB1 proportion = Invaginated laminB1/Total laminB1

B

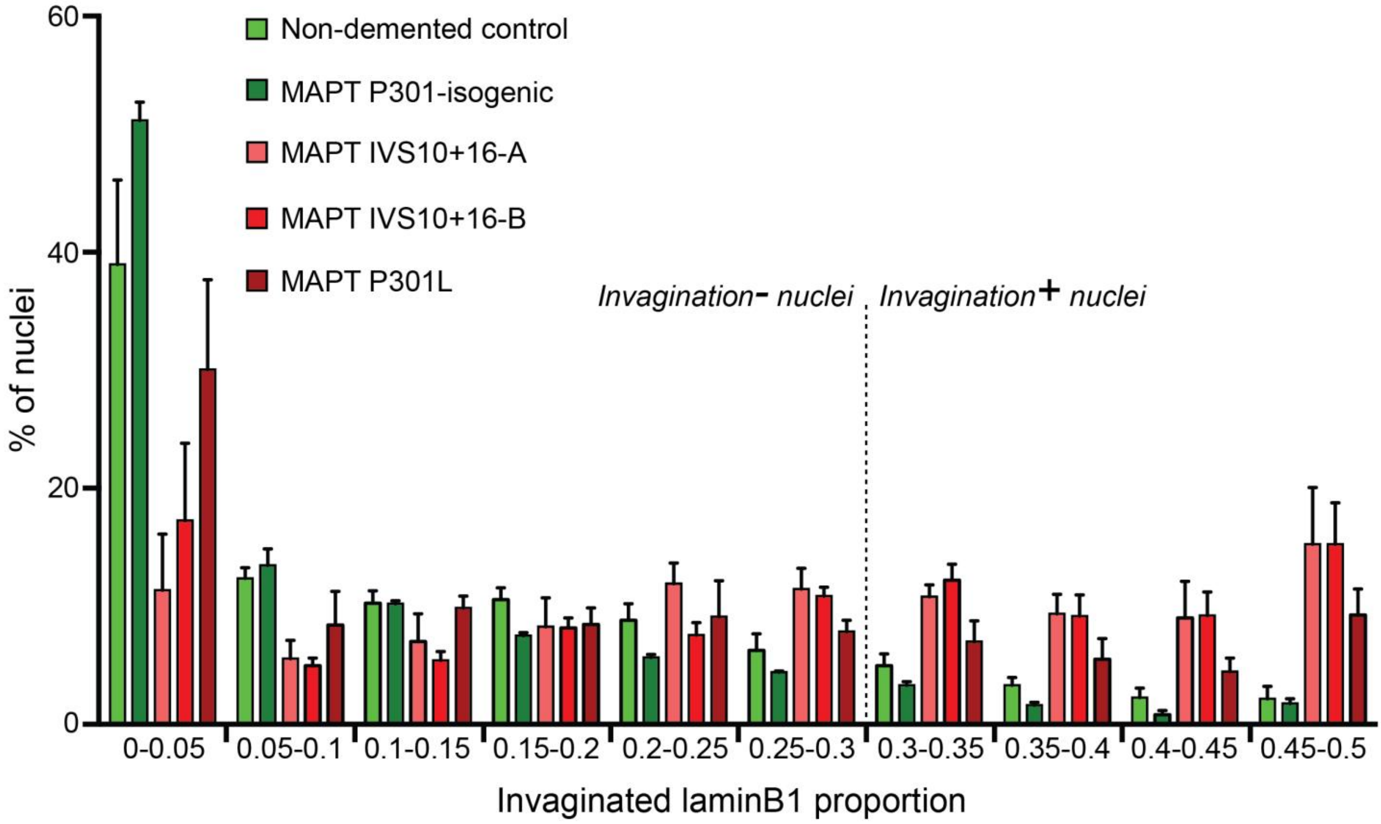

Related to Figure 3; Figure S3. Analysis used to quantify nuclear invaginations in iPSC-derived neurons.

(A) Immunofluorescence of iPSC-derived neurons (*MAPT* P301L; 120DIV), left panel shows LaminB1 (black) and right panel shows processing algorithm performed using the Fiji software. Distinct nuclei are identified by yellow boundaries. LaminB1 signal was assigned as either proximal to the nuclear boundary (cyan) or invaginated (i.e., within the nucleus; magenta). The invaginated laminB1 proportion is calculated from the invaginated laminB1 divided by the total laminB1 signal per nucleus. **(B)** Percentage of nuclei with binned invaginated laminB1 proportions (0.05 intervals) was used to threshold nuclei that are positive for invagination. A distinct population is evident >0.25 invaginated laminB1, this was assigned as invagination positive.

Figure S4

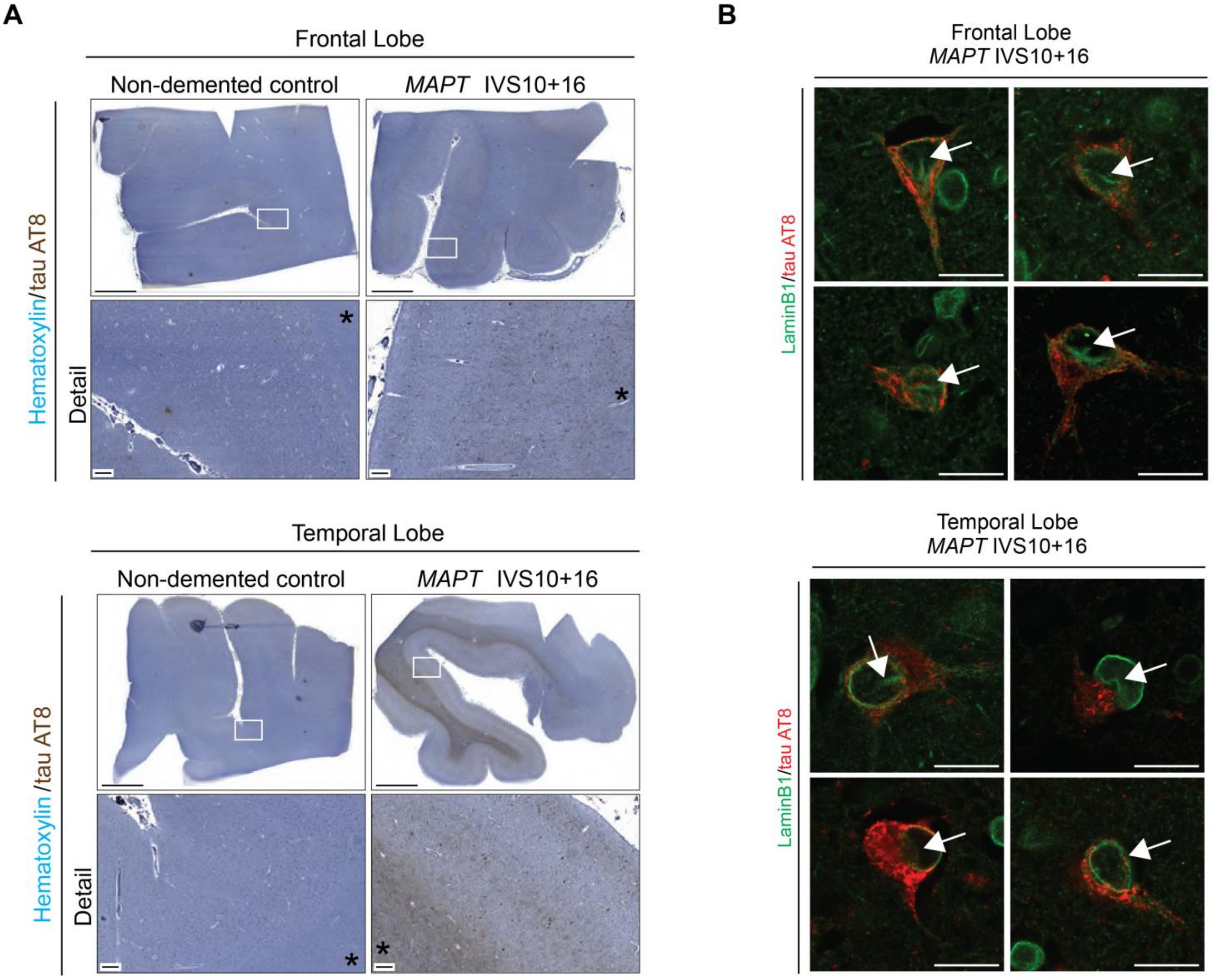

A
Frontal Lobe
Non-demented control
MAPT IVS10+16
Hematoxylin/tau AT8
Detail
*
*
Temporal Lobe
Non-demented control
MAPT IVS10+16
Hematoxylin /tau AT8
Detail
*
*
B
Frontal Lobe
MAPT IVS10+16
LaminB1/tau AT8
Temporal Lobe
MAPT IVS10+16
LaminB1/tau AT8

Related to Figure 5; Figure S4. Phosphorylated disease associated tau is present in *post-mortem* sections from frontal and temporal lobe of FTD-*MAPT*.

(A) 3,3′-Diaminobenzidine (DAB) staining for phosphorylated tau AT8 (brown) in frontal and temporal lobe of subjects carrying MAPT IVS10+16 mutation or non-demented controls (age matched), hematoxylin (blue) was used as nuclear counterstain. Lower panels, detail from white boxes in upper panels. Asterisk indicates white matter position. Scale bars; upper panel = 400 µm, lower panel = 4 µm. **(B)** Confocal images of representative post-mortem neurons from frontal and temporal lobe of subjects carrying MAPT IVS10+16 mutation showing nuclear lamina (green) invaginations (white arrows) in phosphorylated tau (AT8; pS202/pT205; red) containing neurons. Scale bar = 20 µm.

Figure S5

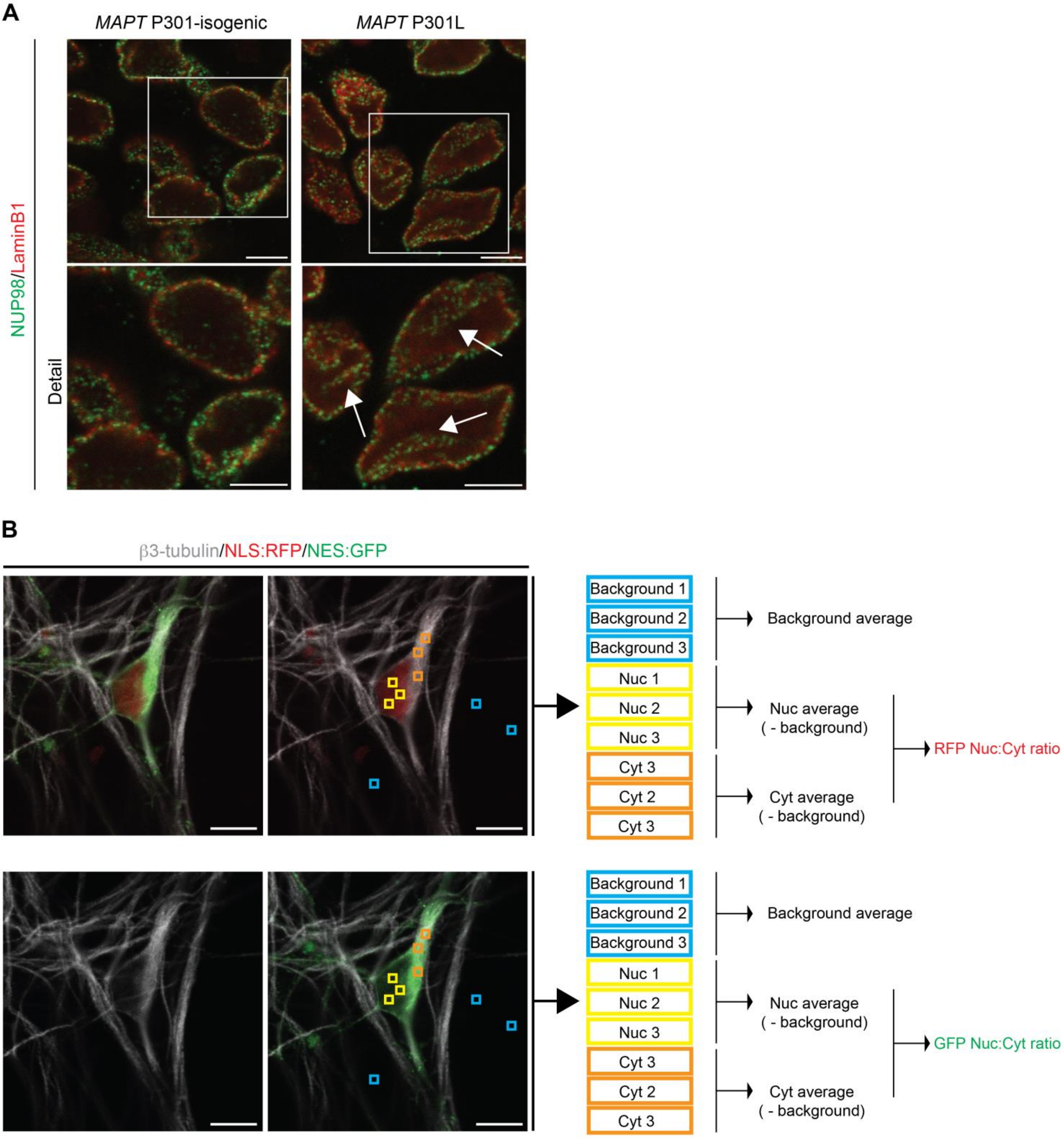

A
MAPT P301-isogenic
MAPT P301L
NUP98/LaminB1
Detail
B
β3-tubulin/NLS:RFP/NES:GFP
Background 1
Background 2
Background 3
Background average
Nuc 1
Nuc 2
Nuc 3
Nuc average
(- background)
Cyt 3
Cyt 2
Cyt 3
Cyt average
(- background)
RFP Nuc:Cyt ratio
Background 1
Background 2
Background 3
Background average
Nuc 1
Nuc 2
Nuc 3
Nuc average
(- background)
Cyt 3
Cyt 2
Cyt 3
Cyt average
(- background)
GFP Nuc:Cyt ratio

Related to Figure 6; Figure S5. Analysis of functional assay demonstrating disruption of nucleocytoplasmic transport in human iPSC-derived FTD-MAPT neurons.

(A) Nuclear pores are clustered within nuclear membrane invaginations in FTD-MAPT neurons. Nuclear pore subunit NUP98 (green) remains co-localised with laminB1 (red) within nuclear invaginations in *MAPT* P301-isogenic neurons and *MAPT* P301L, (120 DIV). Arrows indicate nuclear membrane invaginations. Scale bars = 5 µm. **(B)** Left panels, immunofluorescence of iPSC-derived neurons (*MAPT* P301-isogenic; 120DIV), showing β3-tubulin (grey), NLS:RFP (nuclear localisation signal fused to red fluorescent protein; red) and NES:GFP (nuclear export signal fused to green fluorescent protein; green), individual panels show combinations of signals described above from the same field. Scale bar = 10 µm. To establish the efficiency of nucleocytoplasmic transport in FTD-MAPT neurons, we calculated the ratios of NLS:RFP and NES:GFP inside nucleus and within the cytoplasm. Background (Blue), Nuclear (Nuc; yellow) and cytosolic (as determined by β3-tubulin signal; Cyt; orange) boxed areas were randomly selected and intensity of signal quantified. After background subtraction, intensity values for the nucleus and cytosol were averaged and a ratio established. Four different experiments were performed on each genotype (*MAPT* IVS10+16-A/B, *MAPT* P301L, non-demented control and MAPT P301-isogenic control) with at least six neurons per genotype.
